# Effectiveness of photobiomodulation therapy in improving health indicators in obese patients: a systematic review and meta-analysis of RCTs

**DOI:** 10.1186/s12906-025-04874-2

**Published:** 2025-04-11

**Authors:** Wenjuan Sun, Zexiang Zhuang, Li Yang, Jie Zhou, Linan Zhang, Junhua Yuan

**Affiliations:** 1 Health Management Center, Shenzhen Qianhai Shekou Free Trade Zone Hospital, Shenzhen, China; 2 Gariatric Ward, Shenzhen Qianhai Shekou Free Trade Zone Hospital, Shenzhen, China; 3 Rehabilitation Department, Peking University Shenzhen Hospital, Shenzhen, China; 4 Nursing Department, Shenzhen Qianhai Shekou Free Trade Zone Hospital, Shenzhen, China; 5 Department of Special Medicine, School of Basic Medicine, Qingdao University, Qingdao, China

**Keywords:** Low-level laser therapy, Photobiomodulation, Obesity, Meta-analysis, Anthropometrics, Metabolism

## Abstract

**Objective:**

This meta-analysis is aimed to verify the effectiveness and safety of Photobiomodulation (PBM) on body measurements, metabolic indicators, and inflammation indicators in obese patients across randomized controlled trials with various comparators.

**Methods:**

From the inception of databases to January 5, 2025, we conducted a comprehensive literature search across PubMed, OVID, Cochrane, Embase, Web of Science, LILACS, Chinese Scientific Journals Database (VIP), Wanfang and China National Knowledge Infrastructure (CNKI). Two reviewers independently performed the search, extracted data, and assessed study quality based on predefined inclusion and exclusion criteria. Data analysis was carried out using Review Manager 5.4 software. The reporting and quality assessment of this review study was guided by the PRISMA and AMSTAR.

**Results:**

Eleven RCTs with a total of 569 patients were included in meta-analysis. The pooled data revealed that PBM demonstrated significantly improvements in body anthropometric measurements, such as waistline [MD = - 7.28, 95% CI (- 9.97 to - 5.67), *p* < 0.00001], weight [MD = - 3.54, 95% CI (- 5.97 to - 1.11), *p* < 0.00001], BMI [MD = - 1.18, 95% CI (- 1.93 to - 0.43), *p* = 0.002]. PBM also showed potential in the reduction of CRP [MD = - 0.99, 95% CI (- 1.17 to - 0.82), *p* < 0.00001], as well as in TC, and HOMA-IR, which is [MD = - 23.01, 95% CI (- 31.68 to - 14.35), *p* < 0.00001] and [MD = - 0.46, 95% CI (- 0.73 to - 0.20), *p* = 0.0007] respectively. No significant differences were found in reducing WHR [MD = - 0.05, 95% CI (- 0.1 to 0.00), *p* = 0.05], fat mass percentage [MD = - 0.28, 95% CI (- 1.25 to 0.69), *p* = 0.57] and insulin [MD = - 1.98, 95% CI (- 4.20 to 0.23), *p* = 0.08].

**Conclusion:**

The results of our study suggest that PBM may offer potential benefits for treating obesity, showing some improvements in key indicators such as BMI, weight, waist circumference, CRP, TC, and HOMA-IR compared to exercise, dietary changes, and sham PBM. However, further theoretical exploration of PBM is needed, and multi-center, large-scale trials with longer follow-up durations and demographic range are necessary to confirm and validate the findings of our study.

**Registration number:**

CRD42024532988.

**Supplementary Information:**

The online version contains supplementary material available at 10.1186/s12906-025-04874-2.

## Introduction

The prevalence of overweight and obesity is rising alarmingly, in both developed and developing countries worldwide [1]. The ‘Report on Nutrition and Chronic Diseases among Chinese Residents (2020)’ indicates that the overweight and obesity rate among Chinese adults exceeds 50%. By 2030, the adult obesity/overweight rate in China could reach 70%. Obesity is linked to reduced life expectancy, with estimates suggesting a loss of 5 to 20 years, depending on the severity of the condition and the presence of comorbidities [2–4]. A meta-analysis revealed that all causes for mortality increased by 20% in obese patients, reaching up to 200% in morbidly obese patients [5]. The economic burden is also substantial, with 0.7%− 2.8% of the health budget of each country being allocated for the treatment of obesity and its related complications [6].

Therefore, reducing the obesity-related burden to health and societies as well as reversing the increase in obesity prevalence is a high priority worldwide, which is one of the main targets of the ‘Global Action Plan for the Prevention and Control of Noncommunicable Diseases 2013–2020’ [7]. Conventional methods such as enhancing physical activity, dietary adjustments, and medical treatments often achieve limited success. Methods for preventing and treating obesity include dietary adjustments, exercise, lifestyle interventions, medication control, and even surgery. [8]. However, lifestyle interventions require high patient compliance, while surgery and medication control are costly and carry safety risks. Photobiomodulation therapy (PBM) has emerged as a potential complementary therapeutic strategy to address this issue. PBM biomodulates cellular functions by irradiating specific tissues with light energy, promoting tissue repair and alleviating inflammation. It has been utilized to manage metabolic conditions by increasing the production of lipoprotein lipase, releasing vasodilators, and improving microcirculation. Furthermore, it may reduce the risk of metabolic complications and comorbidities associated with obesity by enhancing anti-inflammatory mediators, reducing fat accumulation, and lowering serum lipid levels [9–11]. Though there has been some high quality RCTs published within our search scope, there is controversy among different studies regarding the effects of PBM on obesity-related indicators. Therefore, we searched the existing high-quality RCTs to conducte a meta-analysis and evaluate the effectiveness and safety of PBM in treating obesity in anthropometrics, metabolism, and inflammation indicators. We aim to provide a research basis for the clinical application of PBM.

### Methods

The search procedures adhered to the guidelines recommended by the Preferred Reporting Items for Systematic Reviews and Meta-Analysis (PRISMA) [12] as well as the Assessing the Methodological Quality of Systematic Reviews 2 (AMSTAR 2) [13]. The PRISMA checklist of this study is provided in Supplementary Material S1. This study quality was evaluated as high according to AMSTAR 2, which can be seen in Supplementary Material S2. The registration number of this study is CRD42024532988 in PROSPERO (https://www.crd.york.ac.uk/prospero/). The ethical approvel was not necessary because no primary private data was used.

### Search strategy

We conducted a systematic literature search from 8 databases inception to January 5, 2025, including PubMed, OVID, Cochrane, Embase, Web of Science, LILACS, Chinese Scientific Journals Database (VIP), Wanfang and China National Knowledge Infrastructure (CNKI). MeSH terms and key terms were combined to search as following: ('Obesity'[Mesh] OR'Obesity Abdominal'[Mesh] OR'Overweight'[Mesh]) AND ('Low-Level Light Therapy'[Mesh]); (Obesity OR Overweight OR'Obese patient*'OR'metabolic syndrome') AND ('low-level laser therapy'OR'Low Level Light Therapy'OR'low-level light therapy'OR'Photobiomodulation Therap*'Photobiomodulation*'OR'Laser Biostimulation'OR'Light-Emitting Diode'). The detail of search strategy is available in Supplementary Material S3.

We did not limit the language of the articles, and manually searched the reference lists of relevant articles. We also searched related reviews to confirm we didn’t overlook any important studies. Some specific journals were also searched to reconfirm, the journals list can be seen in Supplementary Material S3. Additionally, we searched Google Scholar to minimize the possibility of missing any grey literature. Before conducting data analysis, we re-ran the search strategy to ensure that all relevant literature was included. No additional literature was found.

### Inclusion and exclusion criteria

Consideration for inclusion and exclusion criteria can be approached from five aspects, including study type, study population, experimental group intervention, control group intervention, and outcome reporting.

The inclusion criterias were: (1) study design was RCT; (2) population was obesity, overweight, or aiming to lose weight; (3) intervetnion of experimental group should be PBM; (4) the control group should be exercise or diet or sham PBM; (5) the outcomes are weight, BMI (Body mass index), waistline, fat mass percentage, WHR (Waist-to-hip ratio), TC (Total cholesterol), HOMA-IR (Homeostasis model of assessment for insulin resistence index), Insulin, and CRP (C-reactive protein). The study included must report at least one outcome of those. The presentation of outcome measures should include mean and standard deviation values.

The exclusion criterias were: (1) quasi-experimental study, case reports, reviews, editorials, comments, pilot study or study protocols; (2) interventions of experimental group were high intensity laser therapy or PBM combined with acupunture; (3) interventions of control group were medicine or other other complementary methods; (4) animal studies; (5) studies lacking sufficient data to calculate the mean and standard deviation of post intervention..

### Literature screening

We use EndNote 21 to manage importing literatures. After the two reviewers (WJ. S and ZX. Z) independently conducted deduplication, they proceed to read the titles and abstracts of all articles according to predefined standard lable rules (‘TBC’ mean ‘To Be Confirmed’ indicating the need to read the full text to determine inclusion,"Not related design"indicated not meeting RCT study design, et al.). Subsequently, they read the full article and applied our established inclusion and exclusion criteria to determine whether the article should be included in the meta-analysis. When there was disagreement between the two reviewers, the final decision was made by a senior researcher (JH. Y).

### Data extraction

Two reviewers (WJ. S and ZX. Z) independently extracted data. A standardized data collection form was utilized to summarize the following information, such as the first author, publication year, country, participant characteristics, sample size, study design, intervention details, follow-up duration, and related outcomes. The following outcomes data were collected, weight, BMI, waistline, fat mass percentage, WHR, TC, HOMA-IR, insulin, and CRP. Any discrepancies were resolved through discussion, and in cases where consensus could not be reached, the senior reviewer (JH.Y) made the final decision.

### Quality assessment

Two reviewers (WJ. S and ZX. Z) independently assessed the evidence level (LE) of each selected article based on the criteria outlined by the Oxford Centre for Evidence-based Medicine [14]. The methodological quality of the included studies was evaluated using the Jadad scale (1998) [15], with scores higher than 3 indicating high quality, and scores from 0 to 2 indicating low quality.

Additionally, GRADE criteria was used to assess the quality of evidence [16]. The quality of evidence for meta-analysis results was assessed as very low, low, moderate, or high. The quality of each result can be reduced due to factors such as the risk of bias, imprecision, inconsistency, indirectness, and publication bias.

### Inter-rater reliability analysis

We assessed the inter-rater reliability between the two reviewers (SL and WWA) for trial selection, data extraction, trial quality evaluation, and overall evidence certainty using Cohen's Kappa (κ). The κ values were interpreted based on the following scale: ≤ 0 indicates no agreement; 0.01–0.20 represents slight agreement; 0.21–0.40 corresponds to fair agreement; 0.41–0.60 indicates moderate agreement; 0.61–0.80 reflects substantial agreement; and 0.81–1.00 represents almost perfect agreement [17].

### Risk of bias assessment

Two separate reviewers (WJ. S and ZX. Z) performed a quality assessment independently by employing the Cochrane risk-of-bias tool version 2 (RoB 2) [18]. The RoB 2 Excel Macro Form (Beta Version 7) was utilized to assess the methodological quality of the included studies. We scrutinized the risk of bias across five key areas: (1) bias stemming from the randomization process; (2) bias resulting from deviations from the intended interventions; (3) bias due to missing outcome data; (4) bias in the measurement of the outcome; and (5) bias in the selection of the reported result. The assessment ratings were categorized as (1) low risk of bias, (2) some concerns, or (3) high risk of bias, based on the information and signalling questions provided.

### Statistical analysis

We performed statistical analyses utilizing Review Manager 5.4 software. When more than two articles reported the same result, they were included in the meta-analysis. When dealing with continuous data, the mean difference (MD) and a 95% confidence interval (CI) were used to express effect sizes if the same units were utilized for the same outcome indicators. When different scales or units are used between studies, the standardized mean difference (SMD) and a 95% CI were used. When there is a large difference in sample sizes between studies, or when weighting is needed for different studies, the weighted mean difference (WMD) should be used [19]. When it comes to data integration for subgroups, we followed the methods outlined in the Cochrane Handbook for Systematic Reviews of Interventions [19]. Heterogeneity was evaluated using the *χ*^*2*^ and *I*^*2*^ tests, *I*^*2*^ = 0% led to the use of fixed-effects models. *I*^*2*^>0% indicated potential heterogeneity, leading to the adoption of random-effects models. Sensitivity analysis was conducted by sequentially excluding included studies one by one to assess the robustness of the results and identify sources of heterogeneity [19]. We prospectively conducted subgroup analyses based on follow-up time and different interventions of control group for key outcome indicators, including BMI, weight, and waist circumference. This subgroup analyses not only allowed us to explore sources of heterogeneity, but also enabled us to observe the impact of various influencing factors on outcomes, thereby further validating the credibility of the results.

## Results

### Study selection

**Fig. 1:**
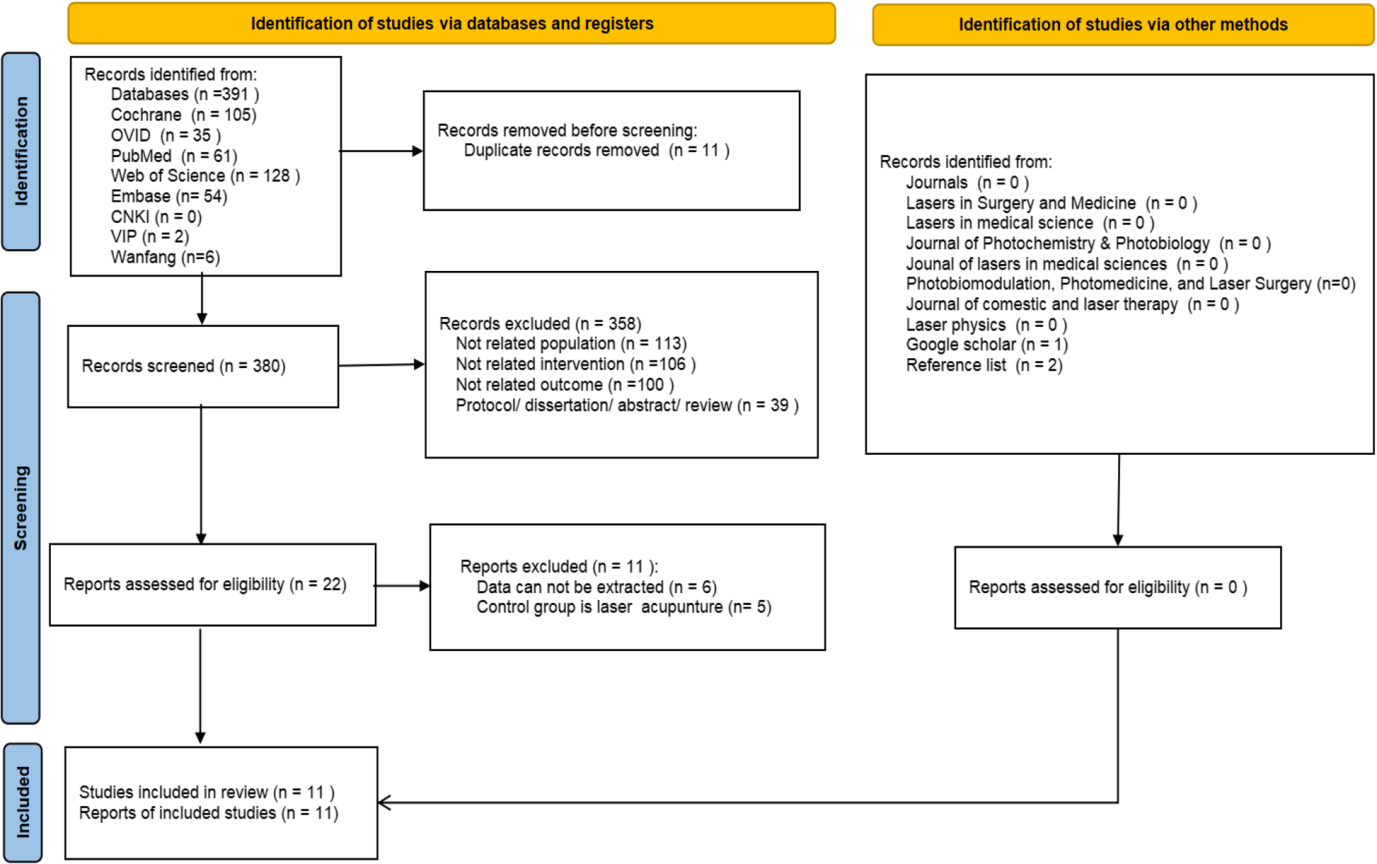
Flow diagram of study selection and identification

According to our search strategy, a total of 345 articles were retrieved. After screening abstracts and full texts and applying inclusion and exclusion criteria, we included 11 RCTs [9–11, 20–27], of which 10 were high-quality RCTs. The literature selection process was conducted using the PRISMA flow diagram, as shown in Fig. 1.

### Study characteristics

Basic characteristics of the included studies were provided in Table 1. The included 11 articles (comprising 569 patients) were all RCTs, with 10 of them rated as high-quality RCTs. The experimental group had 282 participants, while the control group had 287 participants. Patient ages ranged from 14 to 67 years old, treatment and follow-up times varied from 1 to 4 months.

**Table 1:** The characteristics of included studies

Author (year)	Country	LE	Jadad	Study design	Age (Experiment/Control)	Sample size		Intervention		Device and Wavelength	Outcomes	Follow up time	Adverse event	Funding
						**Experiment**	**Control**	**Experiment**	**Control**					
Duarte 2015 [10]	Brazil	1b	5	RCT	(32.20 ± 4.96)/(33.95 ± 4.80)	31	31	LLLT + exercise + diet	exercise + diet	Device: Ga-Al-As semiconductor diode laser; Wavelength: 808 nm Local Irradiation: Abdomen、Gluteus、Quadriceps、Biceps Femoris	①②③④⑤⑦⑧	3 months	NR	CNPq(150,177/2014–3); CNPq 300,654/2013–8; FAPESP (2013/19046–0); FAPESP (2013/04136–4)
Elkablawy 2016 [18]	Egypt	2b	2	RCT	(34.5 ± 2.94)/(34.7 ± 2.94)	20	20	LLLT + exercise	exercise	Device: Lapex 2000 BCS (LipoLaser); Wavelength: NR Local Irradiation: Abdomen	①②③	3 months	NR	No
Elnaggar 2020 [19]	Egypt	1b	4	RCT	(14.27 ± 0.88)/(14.65 ± 1.17)	15	17	LLLT + exercise + diet	exercise + diet	Device: Erchonia® LipoLaser, Erchonia Medical, Inc., McKinney,TX); Wavelength: 635 nm Local Irradiation: Abdomen	③④	1 month	NR	No
Elsayed 2022 [20]	Egypt	1b	5	RCT	(66.10 ± 4.56)/(67.12 ± 5.06)	30	30	LLLT + diet	diet	Device: A non-invasive laser watch; Wavelength: NR System Irradiation: Wrist(wrist acupuncture points)、Nasal cavity	①②③⑧⑨	4 months	NR	No
Elsayed 2023 [9]	Egypt	1b	5	RCT	(68.31 ± 5.17)/(67.35 ± 4.50)	38	38	LLLT + exercise	exercise	Device: A non-invasive laser watch; Wavelength: 650 nm System Irradiation: Wrist(wrist acupuncture points\arteries)、Nasal cavity	①②④⑥⑨	4 months	NR	Te Science, Technology & Innovation Funding Authority (STDF) in cooperation with Te Egyptian Knowledge Bank (EKB)
GamalEldin 2021 [11]	Egypt	2b	3	RCT	(35.05 ± 2.41)/(34.15 ± 2.54)	20	20	LLLT + diet	diet	Device: Low Level Laser (GaAlAs); Wavelength: 680 nm Local Irradiation: Abdomen	①②③	2 months	No adverse event	No
Jackson 2013 [21]	Indian	1b	4	RCT	39.87 (10.01)/39.94 (10.72)	23	29	LLLT	sham laser	Device: Erchonia1 GL Scanner; Wavelength: 532 nm Local Irradiation: Abdomen、back	①②	1 months	No adverse event	No
Mostafa 2016 [22]	Egypt	2b	3	RCT	(14.2 ± 2.11)/(13.9 ± 1.4)	15	15	LLLT + diet	diet	Device:Zerona, Erchonia Corporation, McKinney, TX; Wavelength: 924 nm Local Irradiation: Hips、Things、Waist	①②④	2 months	No adverse event	No
Nagy 2021 [23]	Egypt	1b	4	RCT	(66.10 ± 4.56)/(67.12 ± 5.06)	30	30	LLLT + diet	diet	Device: Abdominal straps containing 4 LED clusters; Wavelength: 660 nm Local Irradiation: Abdomen、Waist	②③⑥	3 months	NR	No
Roche 2016 [24]	USA	1b	4	RCT	(46.32 ± 10.91)/(47.84 ± 9.30)	28	25	LLLT	sham laser	Device: Erchonia Emerald Laser; Erchonia Corporation, McKinney, TX; Wavelength: 532 nm Local Irradiation: Abdomen、Back	①②③	1 month,2 months	No adverse event	No
Sene-Fiorese 2015 [25]	Brazil	1b	4	RCT	(33.06 ± 4.72)/(34.33 ± 4.95)	32	32	LLLT + exercise	exercise	Device: Low Level Laser (GaAlAs); Wavelength: 808 nm Local Irradiation: Abdominal、Quadriceps, Gluteus, Biceps Femoral	①②⑤⑦⑧	3 months	NR	No

*NR* not report

① weight, ② BMI, ③ waistline, ④ WHR, ⑤ fat mass percentage, ⑥ TC, ⑦ Insulin, ⑧ HOMA-IR, ⑨ CRP

### Inter-rater reliability

To evaluate the inter-rater agreement for data extraction, RoB 2, and GRADE assessments, Cohen's Kappa statistic was utilized. The κ values ranged from 0.72 to 0.80, indicating substantial agreement [17].

### Risk of bias and quality assessment

**Fig. 2:**
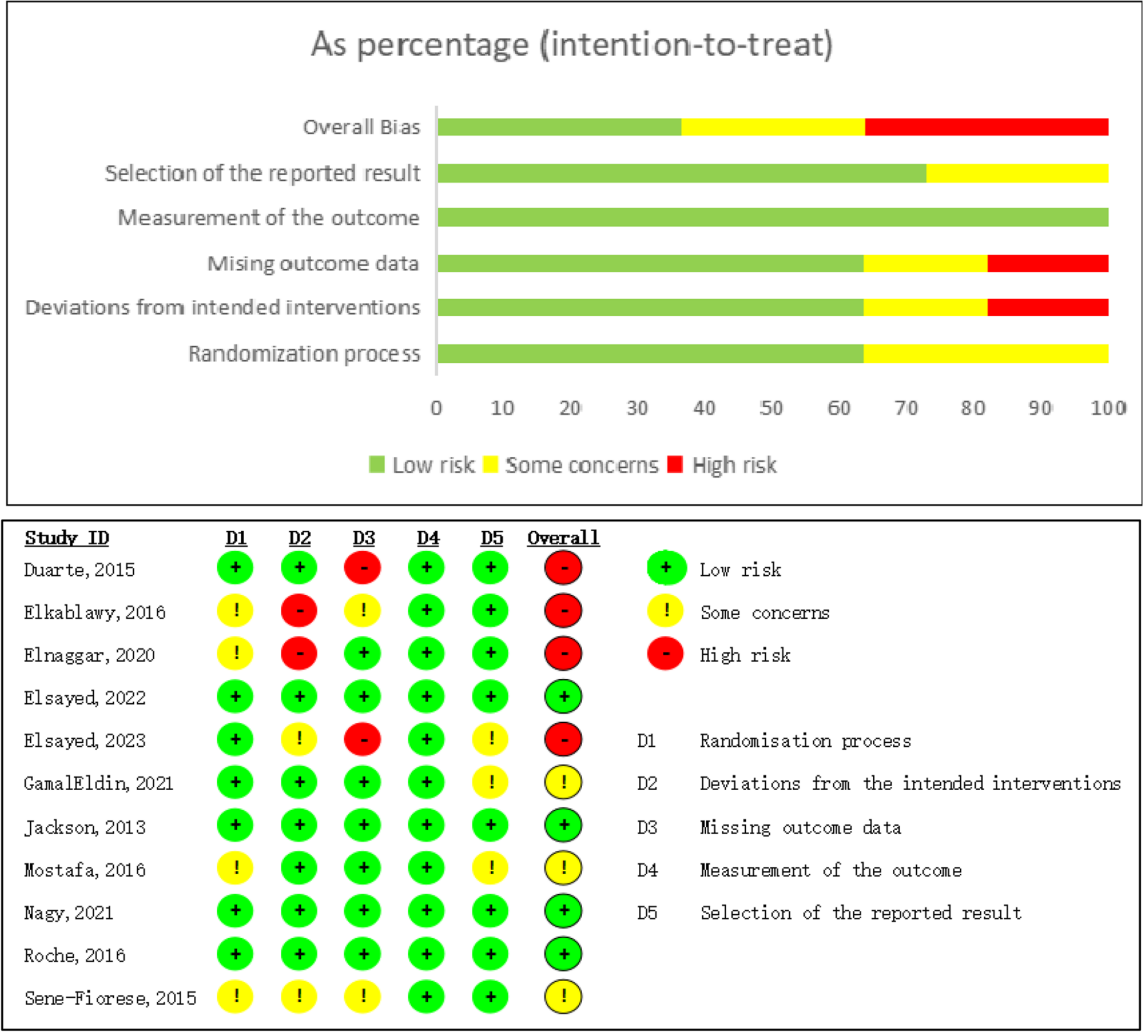
Risk-of-bias graph

The risk of bias assessment was showed in Fig. 2. More than half (63.6%) of the included studies were at low risk of bias concerning the randomization process, deviations from the intended interventions, and missing outcome data. In terms of the measurement of outcomes, all studies were rated as low risk. Regarding the selection of reported results, the majority (72.7%) of the included studies were at low risk of bias. However, in the studies by Elkablawy [20] and Elnaggar [21], there was a high risk of bias in the domain of deviations from the intended interventions, which was attributed to the lack of blinding. In the studies by Duarte [10] and Elsayed [22], there was a high risk of bias in the selection of reported results, likely due to reasons such as participant drop-out or loss to follow-up. Consequently, the overall assessment showed a high-risk percentage of 36.4%, with an additional 27.3% of the studies having some concerns regarding potential bias.

Among the 11 included studies, 10 studies scored 3 or higher on the Jadad scale, indicating high-quality.

### Anthropometric measures

#### BMI

**Fig. 3:**
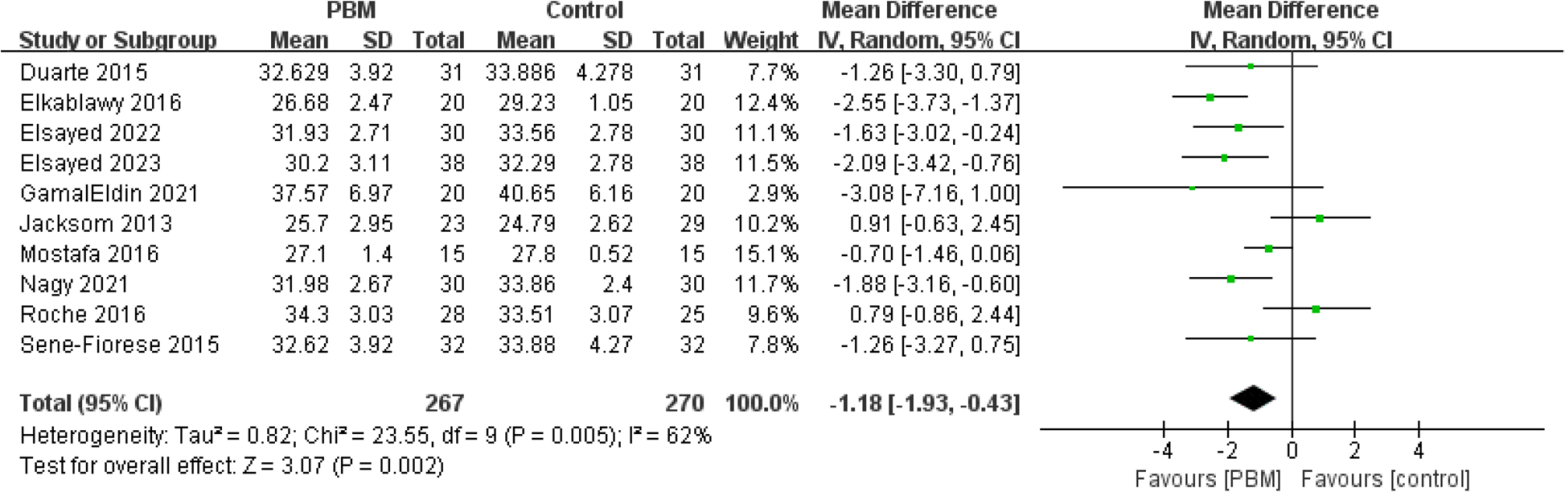
Forest plot of BMI reduction

Ten studies (537 patients) reported data after intervention in BMI [9, 10, 20, 22–27]. Most studies were scored as high quality (Jadad > 3) except Elkablawy 2016. The risk of bias of included studies was low. A random-effect model was used due to a high heterogeneity (*I*^*2*^ = 62%). The combined result showed a significant difference between the two groups [MD = − 1.18, 95% CI (− 1.93 to − 0.43), *p* = 0.002] (Fig. 3).

Sensitivity analysis showed that the pool results were robust, which were provided in Supplementary Material S4. Subgroup analysis of BMI based on different control group interventions and follow-up times yielded consistent results with previous findings. The results of the subgroup analysis were [MD = − 1.18, 95% CI (− 1.93 to − 0.43), *p* = 0.002] and [MD = − 0.99, 95% CI (− 1.75 to − 0.23), *p* = 0.01] respectively. The BMI subgroup analysis forest plot were provided in Supplementary Material S4.

#### Weight

**Fig. 4:**
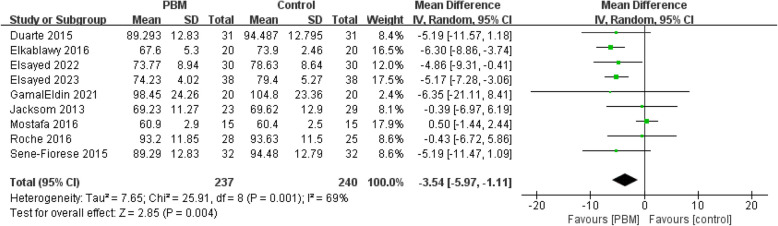
Forest plot of weight reduction

Nine studies (477 patients) reported data after intervention in weight [9–11, 20, 22–24, 26, 27]. Most studies were scored as high quality (Jadad > 3) except Elkablawy 2016. The risk of bias of included studies was low. A random-effect model was used due to a high heterogeneity (*I*^*2*^ = 69%). The combined result showed PBM is more effective in decreasing weight of patients than control group [MD = − 3.54, 95% CI (− 5.97 to − 1.11), *p* = 0.004] (Fig. 4).

Sensitivity analysis was applied due to a high heterogeneity. When we excluded Mostafa (2016), *I*^*2*^ decreased to 0. Mostafa's study's focus on 14–18-year-old adolescents with a small sample size of 15 individuals per group and the lack of statistically significant differences in weight changes between intervention groups could contribute to heterogeneity in the results (*p* = 0.42).The results after sensitivity analysis remained consistent with previous findings [MD = − 5.04, 95% CI (− 6.42 to − 3.67), *p* < 0.00001] (Supplementary Material S5).

Subgroup analysis of weight based on different control group interventions and follow-up times showed consistent results with previous findings. The results of the subgroup analysis were [MD = − 3.54, 95% CI (− 5.97 to − 1.11), *p* = 0.004] and [MD = − 3.25, 95% CI (− 5.55 to − 0.95), *p* = 0.006] respectively. The weight subgroup analysis forest plot were provided in Supplementary Material S5.

#### Waistline

**Fig. 5:**
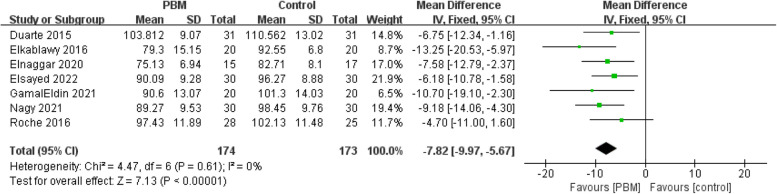
Forest plot of waistline reduction

Seven studies (347 patients) reported data after intervention in waistline [10, 11, 20–22, 25, 26]. Most studies were scored as high quality (Jadad > 3) except Elkablawy 2016. The risk of bias of included studies was low. A fixed-effects model was used due to a very low heterogeneity (*I*^*2*^ = 0%). PBM demonstrates a clear advantage in reducing waist circumference. [MD = − 7.28, 95% CI (− 9.97 to − 5.67), *p* < 0.00001] (Fig. 5).

Sensitivity analysis showed that the pool results were robust, which were provided in Supplementary Material S6. The subgroup analysis on waistline consistently showed clear and significant effects of PBM, aligning with previous findings. Subgroup analysis based on both control group interventions [MD = − 7.82, 95% CI (− 9.97 to − 5.67), *p* < 0.00001] and follow-up times [MD = − 7.47, 95% CI (− 9.49 to − 5.45), *p* < 0.00001] yielded robust results. The waistline subgroup analysis forest plot were provided in Supplementary Material S6.

#### Other outcomes

Due to the limited number of studies reporting WHR, body fat percentage, CRP, TC, insulin, and HOMA-IR, these indicators were summarized and presented in Table 2. The results showed that PBM significantly reduced CRP, TC, and HOMA-IR. However, there were no statistically significant differences between PBM and conventional therapies in terms of WHR, body fat percentage, and insulin. All the forest plots were provided in Supplementary Material S7.

**Table 2:** Meta-analysis results of other outcomes

Outcome	Study included	Population	I^2^	Pooled Effects	*P* value for Z test
WHR [9, 21, 24]	3	138	56%	− 0.05 [− 0.10, 0.00]	0.05
body fat percentage [10, 27]	2	126	0%	− 0.28 [− 1.25, 0.69]	0.57
C- reactive protein [9, 22]	2	136	0%	− 0.99 [− 1.17, − 0.82]	< 0.00001
Total cholesterol [9, 25]	2	136	73%	− 23.01 [− 31.68, − 14.35]	< 0.00001
Insulin [10, 27]	2	126	0%	− 1.98 [− 4.20, 0.23]	0.08
HOMA-IR [10, 22, 27]	3	186	0%	− 0.46 [− 0.73, − 0.20]	0.0007

#### Safety

Out of all 11 articles, 4 mentioned about the adverse event report [11, 23, 24, 26]. Most trials applied a patient self-report and investigator observation method to record and report the adverse events. None of the articles reported adverse events related to PBM.

#### Publication bias

Egger's test was conducted to assess publication bias for the three primary outcomes: weight (t = − 0.40, *p* = 0.6982), waist circumference (t = − 1.30, *p* = 0.2494), and BMI (t = − 0.24, *p* = 0.8156). None of the results showed statistically significant funnel plot asymmetry (*p* > 0.05), indicating no evidence of publication bias (Supplementary Material S8).

#### GRADE assessment

The quality of our results ranged from ‘very low’ to ‘moderate’. The reasons for the downgrade were the flawed methodology of the selected studies, inconsistent results due to significant heterogeneity, imprecise results due to wide confidence intervals and small sample size, and other bias due to publication bias (Table 3).

**Table 3:** Grade assessment results of meta-analysis and quality of evidence

**Outcome**	**No. participants**	**Results**		***I***^***2***^** value**	**Quality of evidence (GRADE)**	**Comments**
		**Relative effect (95% CI)**	**Absolute effects (95%CI)**			
BMI	537	/	MD − 1.18 lower^*^ (− 1.93 to − 0.43)	62	⨁⨁◯◯LOW	Imprecison^a^ Inconsistency^b^
Weight	477	/	MD − 3.54 lower^*^ (–5.97 to − 1.11)	69	⨁⨁◯◯LOW	Imprecison^a^ Inconsistency^b^
Waistline	347	/	MD − 7.28 lower^*^ (− 9.97 to − 5.67)	0	⨁⨁⨁◯MODERATE	Imprecison^a^
WHR	138	/	MD − 0.05 lower^*^ (− 0.1 to 0.00)	56	⨁◯◯◯VERY LOW	Imprecison^a^ Inconsistency^b^ other bias^c^
Fat mass percentage	126	/	MD − 0.28 lower^*^ (− 1.25 to 0.69)	0	⨁◯◯◯VERY LOW	Imprecison^a^ Inconsistency^b^ other bias^c^
CRP	136	/	MD − 0.99 lower^*^ (− 1.17 to − 0.82)	0	⨁⨁◯◯LOW	Imprecison^a^ other bias^c^
TC	136	/	MD − 23.01 lower^*^ (− 31.68 to − 14.35)	73	⨁◯◯◯VERY LOW	Imprecison^a^ Inconsistency^b^ other bias^c^
Insulin	126	/	MD − 1.98 lower^*^ (− 4.20 to 0.23)	0	⨁◯◯◯VERY LOW	Imprecison^a^ Inconsistency^b^ other bias^c^
HOMA-IR	186	/	MD − 0.46 lower^*^ (− 0.73 to − 0.20)	0	⨁⨁◯◯LOW	Imprecison^a^ other bias^c^

*BMI* Body Mass Index, *WHR* Waist-to-hip ratio, *CRP* C-reactive protein, *TC* Total cholesterol, *HOMA-IR* Homeostasis model of assessment for insulin resistance index

^*^Lower values indicated better effect

^a^Small sample or wide confidence intervals

^b^Heterogeneity (*I*^*2*^ > 50%, *p* < 0.05) was found

^c^Publication bias due to funding support

## Discussion

Obesity has reached epidemic levels worldwide over the past 50 years. It has been proven to lead to numerous chronic diseases such as type 2 diabetes, fatty liver disease, hypertension, heart attack, stroke, dementia, osteoarthritis, obstructive sleep apnea syndrome, and more [28]. Throughout the world, numerous dietary and exercise interventions have been developed for obese patients, and several meta-analyses on lifestyle adjustments have demonstrated their effectiveness in short-term weight loss. However, some scholars argued that lifestyle interventions pose a significant challenge in terms of patient adherence, with limited effectiveness and sustainability [28]. This study suggests that PBM may reduce weight, BMI, and waist circumference in obese patients, with more pronounced effects observed in the long term compared to sham, diet and exercise. It also shows potential benefits in reducing inflammation, total cholesterol, and insulin resistance. However, PBM does not demonstrate clear advantages in reducing body fat percentage, WHR, or insulin levels.

### Interpretation of the results

As for anthropometric measures, PBM showed a relatively stronger effect on reducing weight, BMI, and waistline, with more supporting evidence compared to other outcome measures. PBM can reduce BMI, weight, and waistline by 1.18 kg/m^2^, 5.25 kg, and 7.3 cm, respectively. Even after conducting subgroup analysis based on different intervention methods in the control group and varying follow-up durations, this significant difference still persists. This consistency aligns with the results mentioned in the previous laser acupuncture review for treating obese patients [29]. Our meta-analysis further reveals that PBM's effectiveness in reducing waist circumference is more pronounced compared to weight and BMI. PBM proves more effective on reducing waist circumference in obese patients regardless of whether the control group received exercise, dietary interventions, or a combination of both. Since only one study with a sham control was included in the subgroup analysis, the results did not reach statistical significance. However, PBM showed a trend toward reducing waist circumference compared to sham. Moreover, when subgroup analysis based on follow-up duration was performed for waist circumference, PBM consistently demonstrates greater efficacy in reducing waist circumference at any follow-up time point. The reduction in waist circumference with PBM showed statistically significant differences at the first [MD = − 6.40, 95% CI (− 10.43 to − 2.36), *p* = 0.002], second [MD = − 7.03, 95% CI (− 12.76 to − 1.30), *p* = 0.02], third [MD = − 9.17, 95% CI (− 12.45 to − 5.89), *p* < 0.00001], and fourth months [MD = − 6.18, 95% CI (− 10.56 to − 1.80), *p* = 0.006]. This could be because PBM therapy mostly targets the waist area, leading us to predict that PBM may have better effects in patients with abdominal obesity. Caruso-Davis [30] supported our hypothesis by dividing non-obese patients into PBM and sham PBM groups. All patients were required to maintain their dietary habits without significant weight fluctuations (± 1.5 kg) throughout the study period. The results showed a significant reduction in waist circumference in the PBM group compared to the control group after 8 weeks (PBM − 0.78 ± 2.82 cm vs. Placebo 1.35 ± 2.64 cm, *p* < 0.05). Although the subgroup analysis for waist circumference showed good consistency, the subgroup analyses for BMI and weight exhibited inconsistencies. The study found that, compared to diet, exercise, and sham control groups, PBM did not demonstrate consistent advantages in reducing weight and BMI. This inconsistency is not difficult to understand. First, diet and exercise are already well-established weight loss methods. Second, the small sample sizes in each subgroup of the analysis could have contributed to the instability of the results.When conducting subgroup analysis based on follow-up duration for BMI and weight, we observed an interesting phenomenon. In the first and second months, PBM did not show significant differences compared to the control group. However, as the follow-up duration increased, PBM significantly reduced BMI and weight in obese patients in the third and fourth months compared to the control group. This could be because PBM achieves weight loss by altering the body's internal environment and hormone levels, which is consistent with our finding that PBM can reduce insulin resistance.

However, PBM did not show a statistically significant difference in WHR compared to conventional interventions [MD = − 0.05, 95% CI (− 0.1 to 0.00), *p* = 0.05]. This may be due to the high heterogeneity among these three studies and the significant age differences in the study populations. Two studies focused on adolescents under 18 years old, while the third study included individuals over 60 years old, with small sample sizes in all cases. Nevertheless, PBM demonstrates a trend in improving WHR, and future large-scale studies are needed to further confirm its effectiveness. Body fat content is considered to be associated with chronic diseases such as hypertension, especially the increase in abdominal fat distribution, which is highly correlated with cardiovascular and cerebrovascular diseases [31, 32]. Our study found that the PBM group did not show a significant advantage in reducing body fat content compared to the conventional interventions [MD = − 0.28, 95% CI (− 1.25 to 0.69), *p* = 0.57]. It is worth noting that only three studies [10, 11, 27] used body fat percentage as an outcome measure, and the study population was different, which is insufficient to draw conclusions. Furthermore, the mechanism behind PBM-induced fat reduction is not clear. One of the main proposed mechanisms of action is based on the production of transient pores in adipocytes, allowing lipids to leak out [33]. Another mechanism involves the activation of the complement cascade, which could induce adipocyte apoptosis and subsequent release of lipids [34, 35]. Caruso-Davis [30] also found the fat loss was likely due to the laser creating temporary pores in the fat cells, allowing triglycerides to leak out. The leaked triglycerides would then flow back into the lymphatic system, where they might be either utilized as energy through consumption or redistributed. This also resulted in a reduction in total cholesterol (TC) levels in the blood, which has been confirmed in a previous review [34] and is consistent with the results of our meta-analysis. As a result, fat redistribution occurs, yet there is no significant change in overall body fat content. However, there is currently no official consensus on the mechanism behind the fat reduction induced by PBM.

Inflammatory markers are considered closely related to obesity, and in our meta-analysis, we found that PBM can significantly reduce CRP levels [MD = − 0.99, 95% CI (− 1.17 to − 0.82), *p* < 0.00001]. Many studies have investigated the effects of PBM on musculoskeletal pain and inflammatory responses [36–38]. The meta-analysis results indicate that PBM is effective in treating musculoskeletal pain. However, the primary outcome measure in the studies was the VAS pain score, and inflammatory markers such as CRP were not included. Scholars have used PBM for treating rheumatoid arthritis.The results demonstrate a significant reduction in plasma malondialdehyde (MDA), serum nitrate and nitrite levels, serum C-reactive protein (CRP) levels, plasma interleukin- 6 (IL- 6) levels, and glutathione peroxidase (GPx) activity, as well as erythrocyte sedimentation rate (ESR) in patients exposed to laser treatment compared to their pre-treatment levels (*p* < 0.0005) [39]. There is substantial evidence, both mechanistic and clinical, supporting the mechanisms through which PBM modulates inflammatory responses [40–43]. PBM modulates the inflammatory response by regulating the expression of intracellular enzyme cyclooxygenase (COX), affecting the conversion of arachidonic acid (ARA) synthesized from membrane phospholipids to prostaglandins. Specifically, PBM reduces the expression of COX- 2, leading to decreased production of inflammatory mediators such as interleukin- 6 (IL- 6), tumor necrosis factor-alpha (TNF-α), and inducible nitric oxide synthase (iNOS), while increasing the expression of anti-inflammatory mediators like interleukin- 10 (IL- 10) [43]. These mechanisms, observed in various animal models and clinical studies, demonstrate the potential clinical application of PBM in suppressing inflammation.

As for HOMA-IR, PBM also demonstrates clear advantages over conventional intervention methods [MD = − 0.46, 95% CI (− 0.73 to − 0.20), *p* = 0.0007]. Hamblin mentioned that when treating diabetic mice with PBM, both their blood glucose levels and insulin resistance levels significantly decreased. Mice subjected to abdominal irradiation also showed reduced adipose tissue validation levels [44]. That might can explain why PBM can improve insulin resistance. However, the improvement in fasting insulin levels did not reach statistical significance [MD = − 1.98, 95% CI (− 4.20 to 0.23), *p* = 0.08]. This could be due to the fact that the study population did not specifically target diabetic individuals, and the sample size was relatively small. We strongly recommend that future studies expand the trial population of PBM to include patients with obesity accompanied by polycystic ovary syndrome, diabetes, or hypertension, in order to validate its effects on metabolic markers related to chronic diseases.

### Strengths and limitations

This systematic review faced several constraints. The included RCTs had relatively small sample sizes and exhibited clinical and methodological diversity, including variations in patient age, obesity severity, power and density differences, types of control groups, and assessed outcomes. Most studies were conducted in Brazil and Egypt, which may introduce geographical bias. Although blinding PBM therapists is inherently challenging, efforts should be made to blind patients, other healthcare providers, and outcome assessors to minimize performance and assessment biases. Another notable limitation is the short follow-up duration, as none of the studies exceeded six months, whereas long-term effects are critical for practical obesity interventions. Additionally, while all studies reported using PBM (wavelength range 600–1000 nm), the lack of standardization in treatment parameters remains a significant issue. Research using consistent and agreed-upon parameters is almost non-existent, highlighting the need for consensus among experts. Despite these limitations, this study represents the first meta-analysis examining PBM's efficacy in obesity treatment. The quality of the literature included in this review study is generally high, and a comprehensive range of indicators was considered. Furthermore, this study strictly adhered to the PRISMA and AMSTAR 2 guidelines for reporting and quality assessment. All relevant research data were made publicly available to ensure transparency, replicability, and rigour for peer review. This study provides valuable insights for clinical decision-making on PBM use, but population differences should be considered in clinical applications, as most included studies were from Egypt and Brazil. In summary, future research should focus on large sample sizes, diverse countries and demographics, multi-center studies, extended follow-up durations, and the inclusion of inflammation and metabolic indicators. Trial design and reporting should strictly adhere to the CONSORT statement [44]. Finally, we hope to see more research exploring the mechanisms of PBM to uncover the scientific principles underlying its effects on weight loss.

## Conclusion

This review suggests that PBM may be a safe and effective treatment for obesity compared to sham and conventional interventions, with the most significant effect observed in reducing waist circumference. PBM also shows advantages over sham and conventional interventions in reducing BMI and weight, particularly with more pronounced effects over longer follow-up periods. However, the potential benefits of PBM in reducing CRP, TC, and HOMA-IR require further investigation.

## Supplementary Information

Supplementary Material 1. S1. PRISMA 2020 checklist.

Supplementary Material 2. S2. AMSTAR 2 checklist.

Supplementary Material 3. S3. Search strategy and specific journals list.

Supplementary Material 4. S4. BMI subgroup and sensitivity analysis.

Supplementary Material 5. S5. Weight subgroup and sensitivity analysis.

Supplementary Material 6. S6. Waistline subgroup and sensitivity analysis.

Supplementary Material 7. S7. Other outcomes analysis.

Supplementary Material 8. S8. Publication bias.

## Data Availability

Data is provided within the manuscript or supplementary information files. More detail can contact the corresponding author.

## Abbreviations

PBM: Photobiomodulation therapy
RCT: Randomized Controlled Trial
PRISMA: Preferred Reporting Items for Systematic Reviews and Meta-Analysis
AMSTAR 2: Assessing the Methodological Quality of Systematic Reviews 2
BMI: Body Mass Index
WHR: Waist-to-hip ratio
TC: Total cholesterol
HOMA-IR: Homeostasis model of assessment for insulin resistance index
CRP: C-reactive protein
TBC: To Be Confirmed
LE: Levels of Evidence
MD: Mean difference
CI: Confidence interval
SMD: Standardized mean difference
WMD: Weighted mean difference
