# Supplementary material for: Effectiveness of photobiomodulation therapy in improving health indicators in obese patients: a systematic review and meta-analysis of RCTs
Source: BMC Complement Med Ther. 2025 Apr 11;25:133. doi: 10.1186/s12906-025-04874-2 (PMC11992763; doi:10.1186/s12906-025-04874-2)

| **Item** | **Mesh terms** | **Key terms** |
| --- | --- | --- |
| **Population** | ("Obesity"[Mesh]OR "Obesity, Abdominal"[Mesh]OR "Overweight"[Mesh] ) | “Obesity”OR “Excessive weight”OR“Overweight”OR “Obese condition”OR “Obese state”OR “Morbid obesity”OR “Adiposity”OR “Unhealthy weight” OR "Abdominal Obesities"OR"Obesities, Abdominal"OR"Central Obesity"OR"Central Obesities"OR"Obesities, Central"OR"Obesity, Central"OR"Abdominal Obesity"OR"Obesity, Visceral"OR"Visceral Obesity"OR"Obesities, Visceral"OR"Visceral Obesities"OR “metabolic syndrome” |
| **Intervention** | "Low-Level Light Therapy"[Mesh] | “low-level laser therapy”OR “Low Level Light Therapy”OR “low-level light therapy”OR “Photobiomodulation Therap*”OR “Low-Power Laser Therapy”OR “Photobiomodulation*” OR “Laser Biostimulation” OR “Laser Phototherapy” “Low-Power Laser Irradiation” OR LLLT OR LED OR “Light-Emi  tting Diode” |

**Supplementary Material S3 Search Strategy and journal list**

**Specific journal we searched:**

Lasers in Surgery and Medicine

Lasers in medical science

Journal of Photochemistry & Photobiology

Jounal of lasers in medical sciences

Photobiomodulation, Photomedicine, and Laser Surgery

Journal of comestic and laser therapy

Laser physics

**Search strategy of all databases**

**LILAS:**

(low-level laser therapy) OR (Low Level Light Therapy) OR (low-level light therapy) OR (Photobiomodulation Therap*) OR (Low-Power Laser Therapy) OR (Photobiomodulation*) OR (Laser Biostimulation) OR (Laser Phototherapy) OR (Low-Power Laser Irradiation) OR (LLLT) OR (PBM) OR (Light-Emitting Diode) OR (LED)

(Obesity) OR (Excessive weight) OR (Overweight) OR (Obese condition) OR (Obese state) OR (Morbid obesity) OR (Adiposity) OR (Unhealthy weight) OR (Abdominal Obesities) OR (Obesities, Abdominal) OR (Central Obesity) OR (Central Obesities) OR (Obesities, Central) OR (Visceral Obesity) OR (metabolic syndrome)


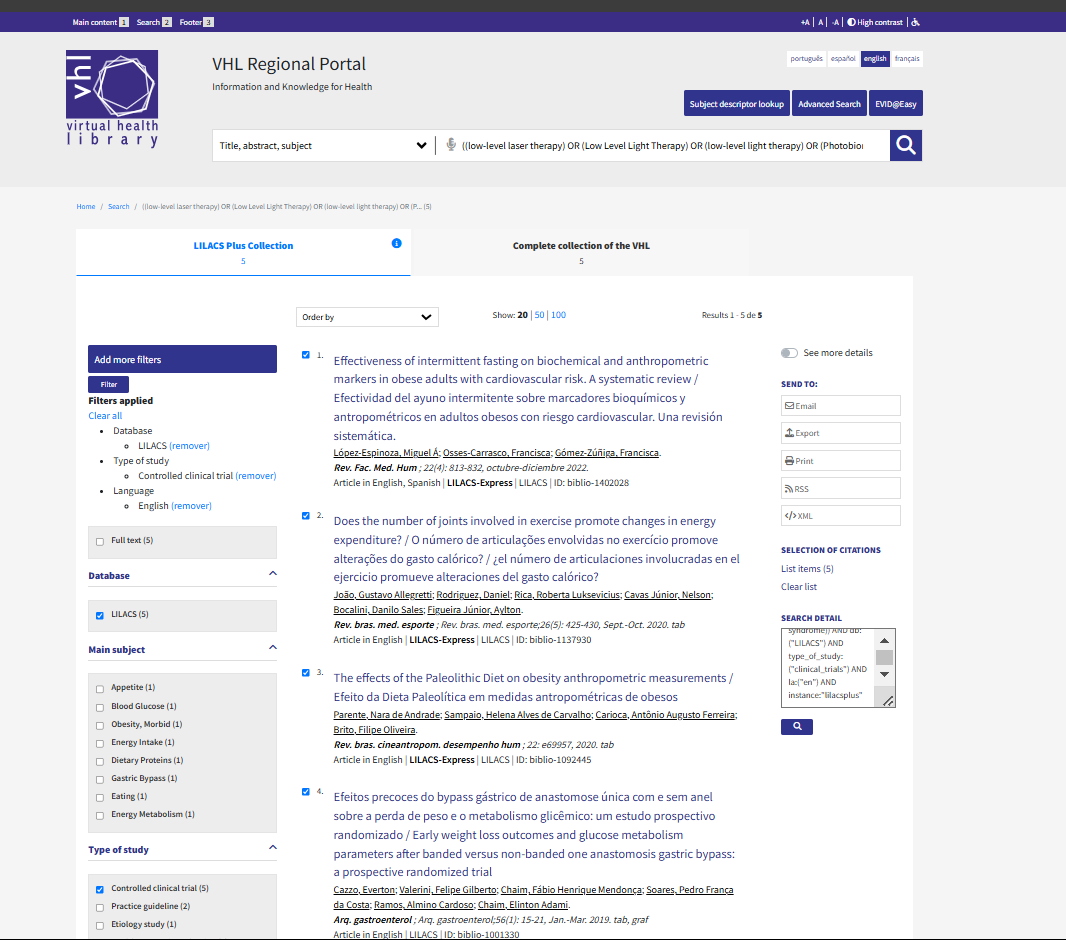


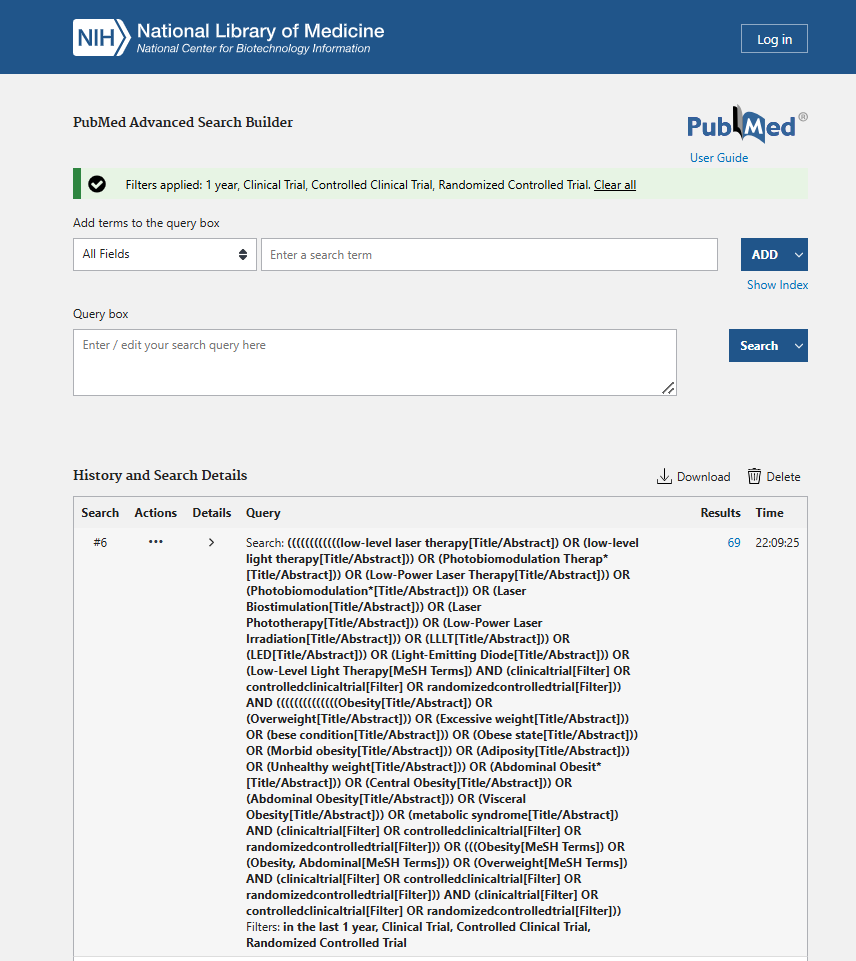

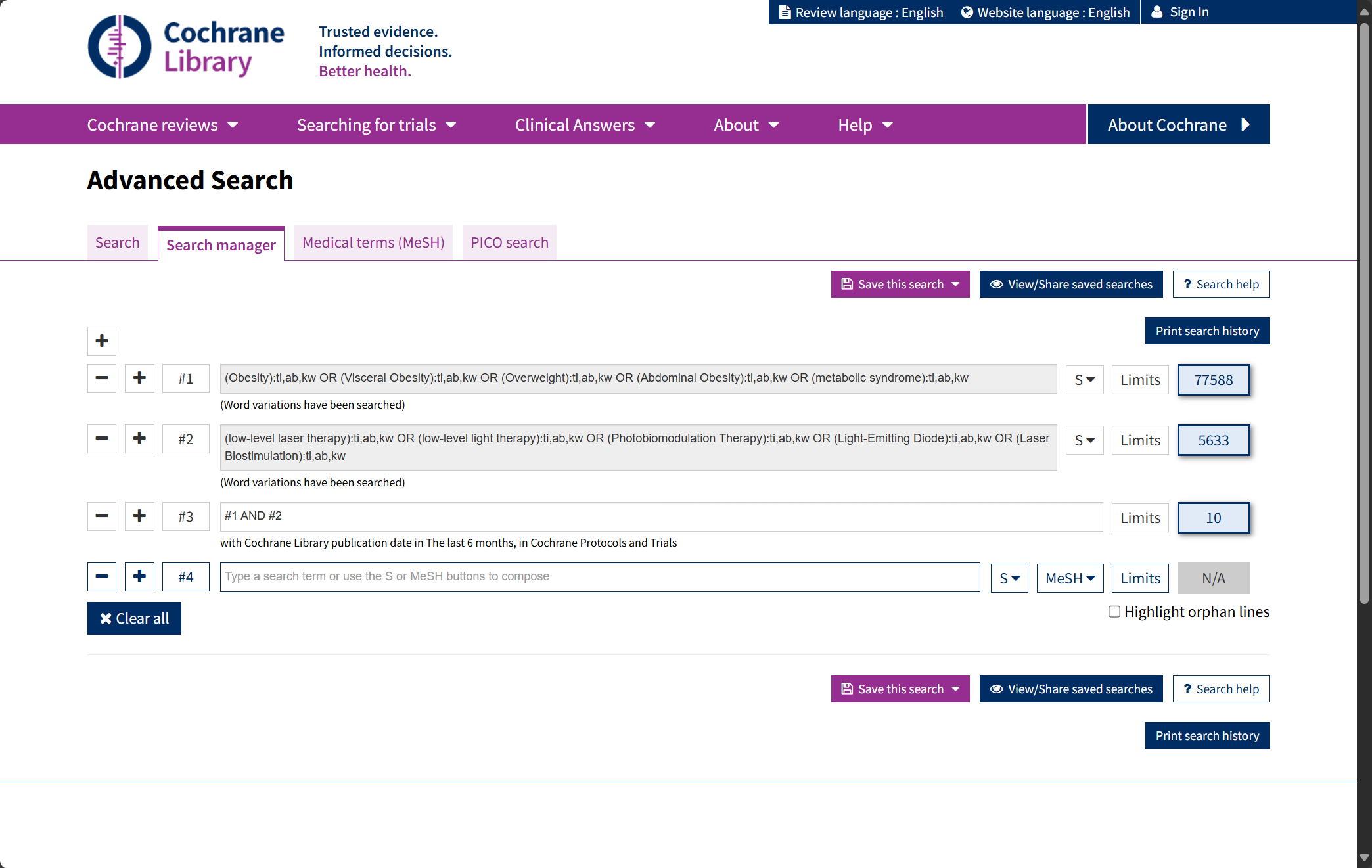

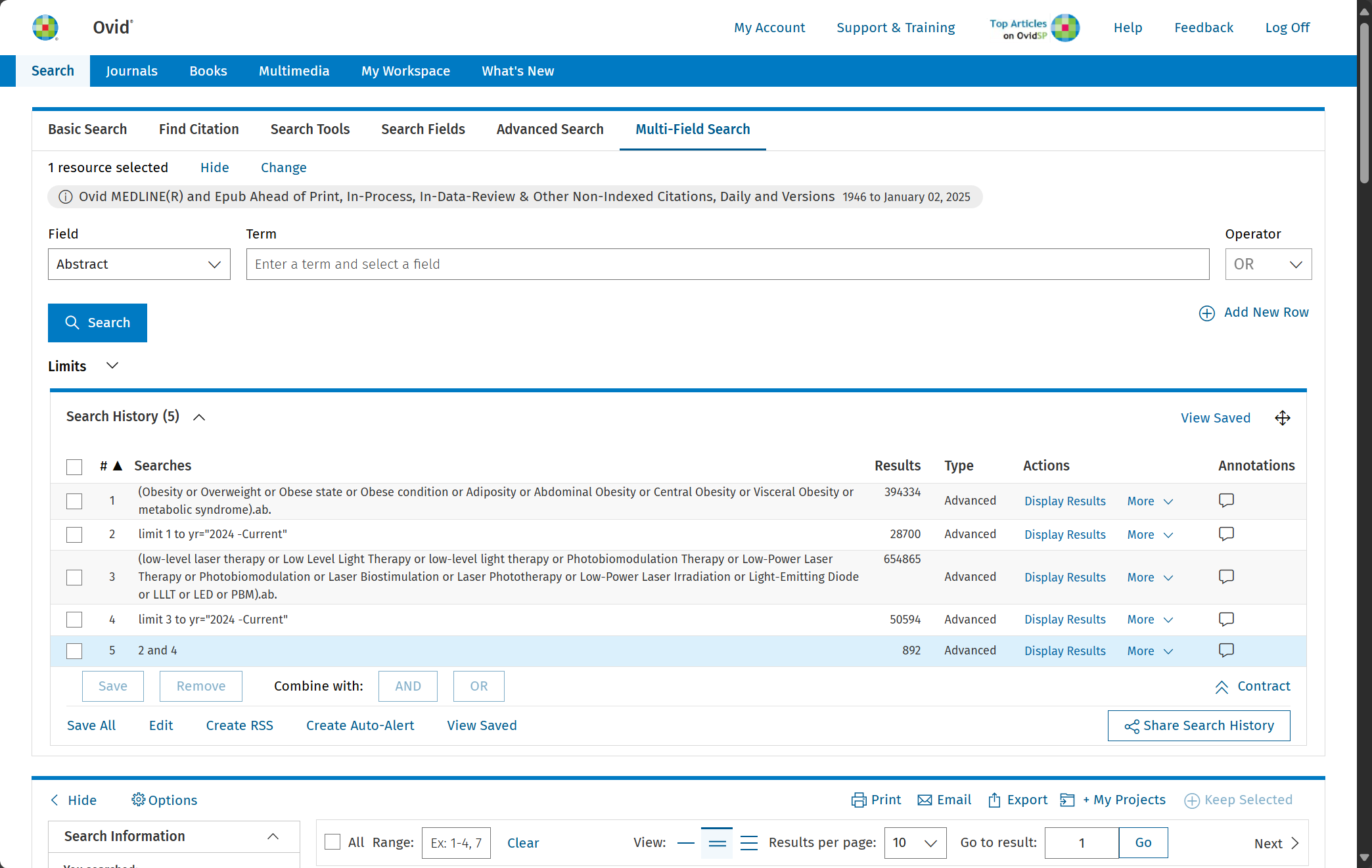

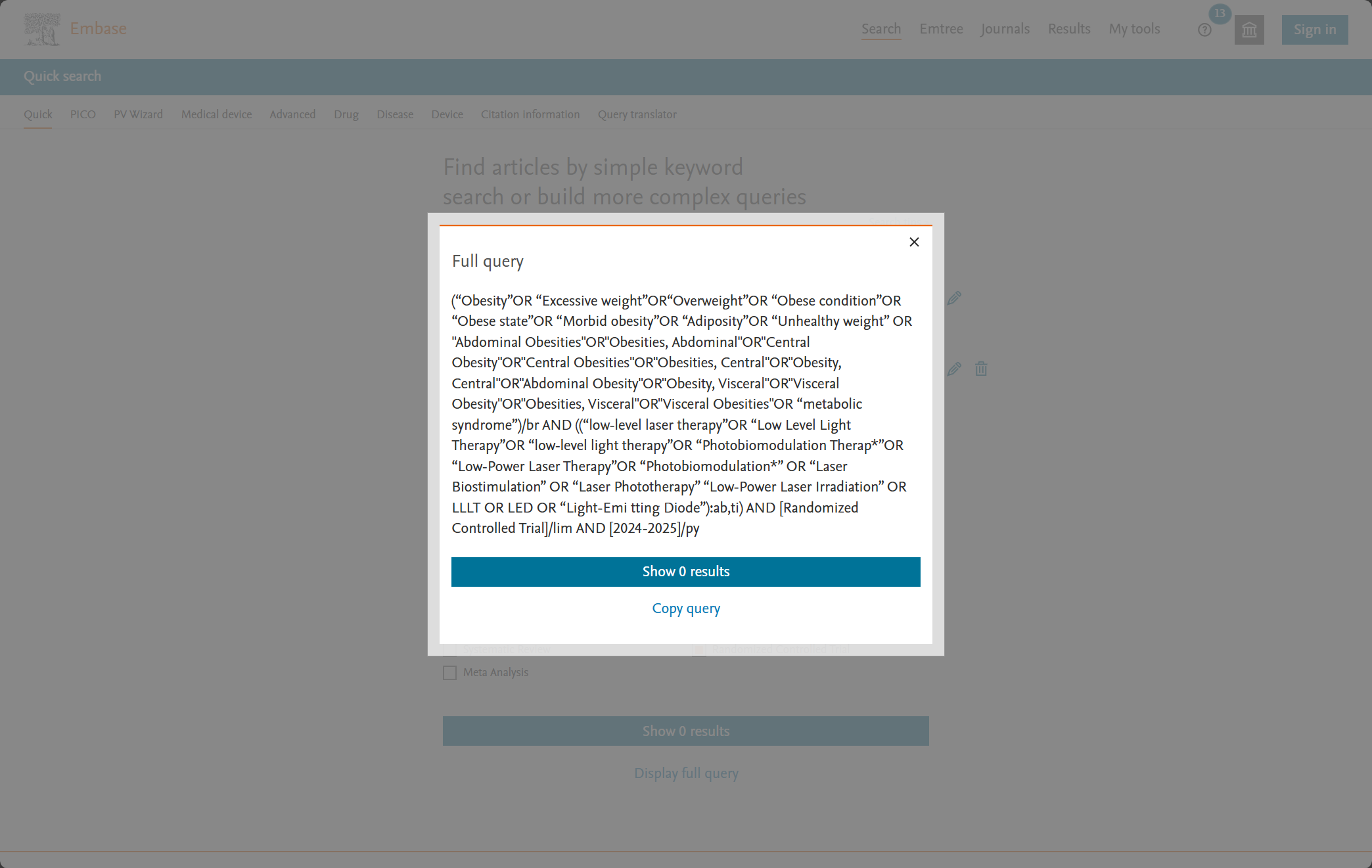

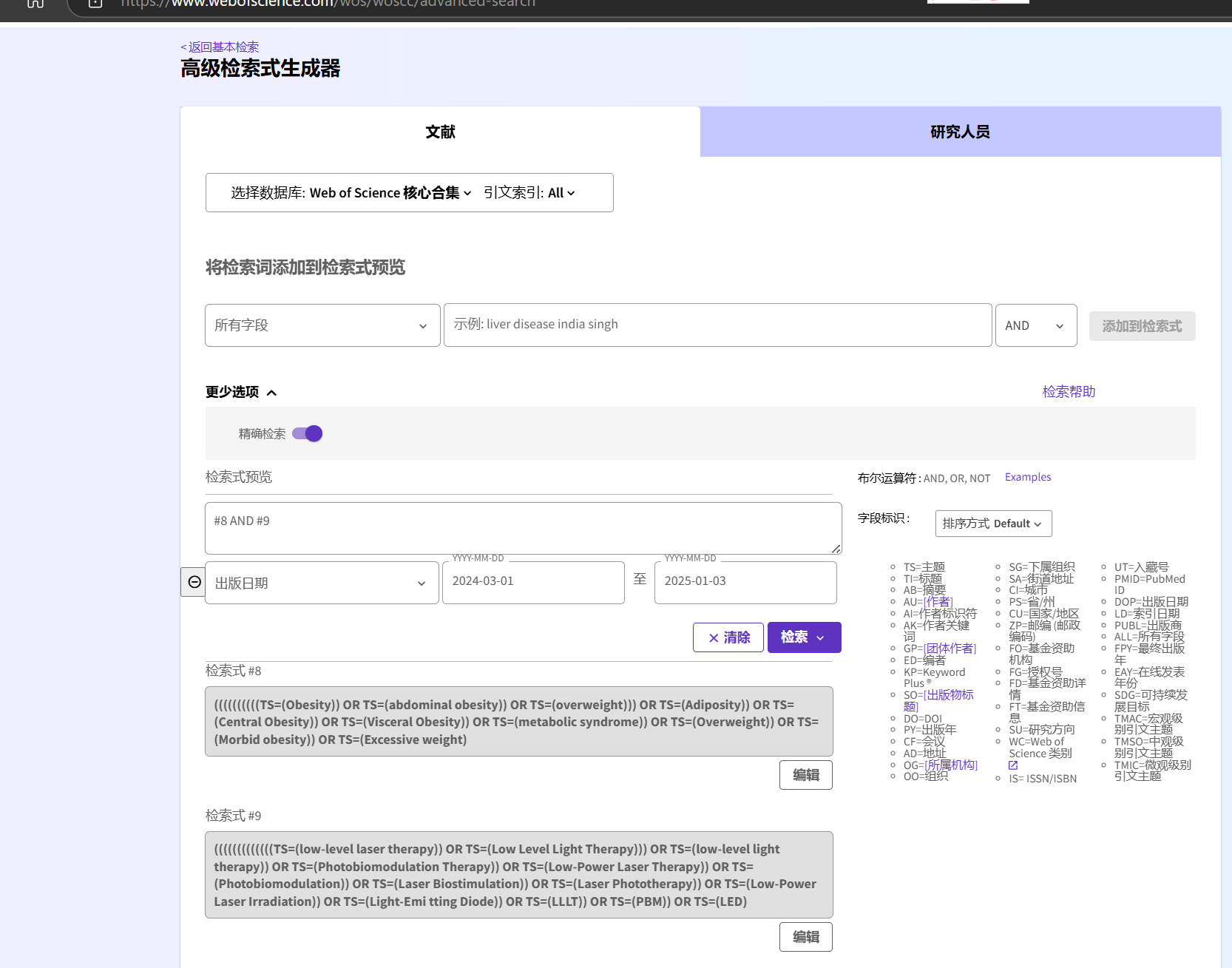

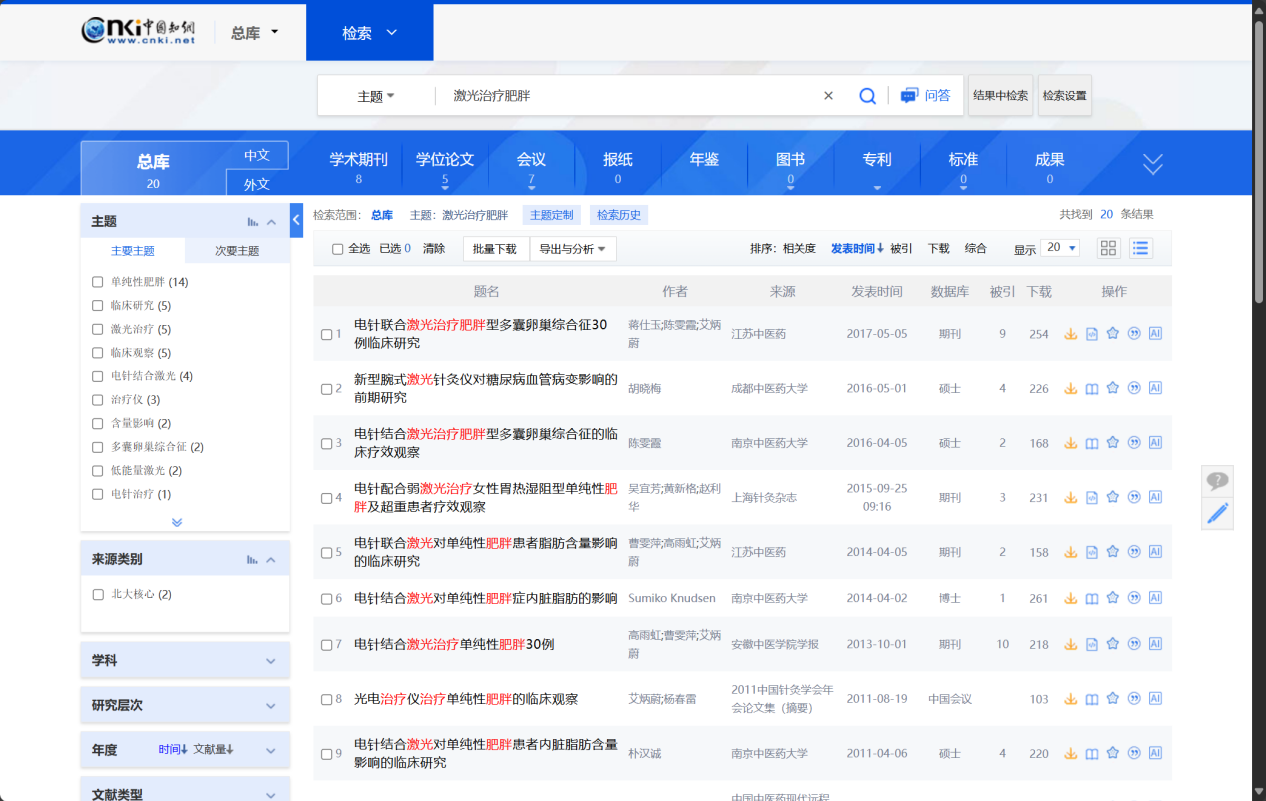

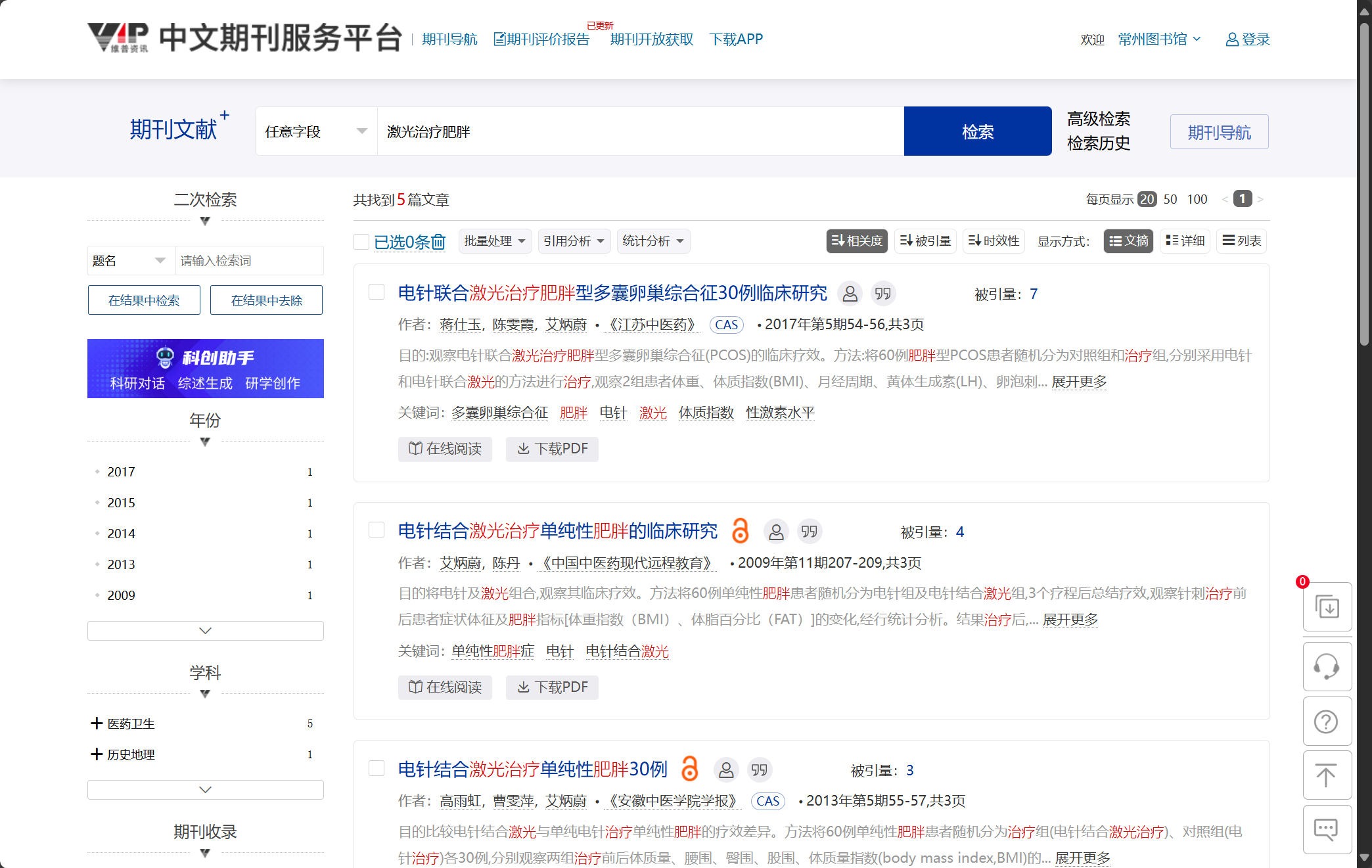

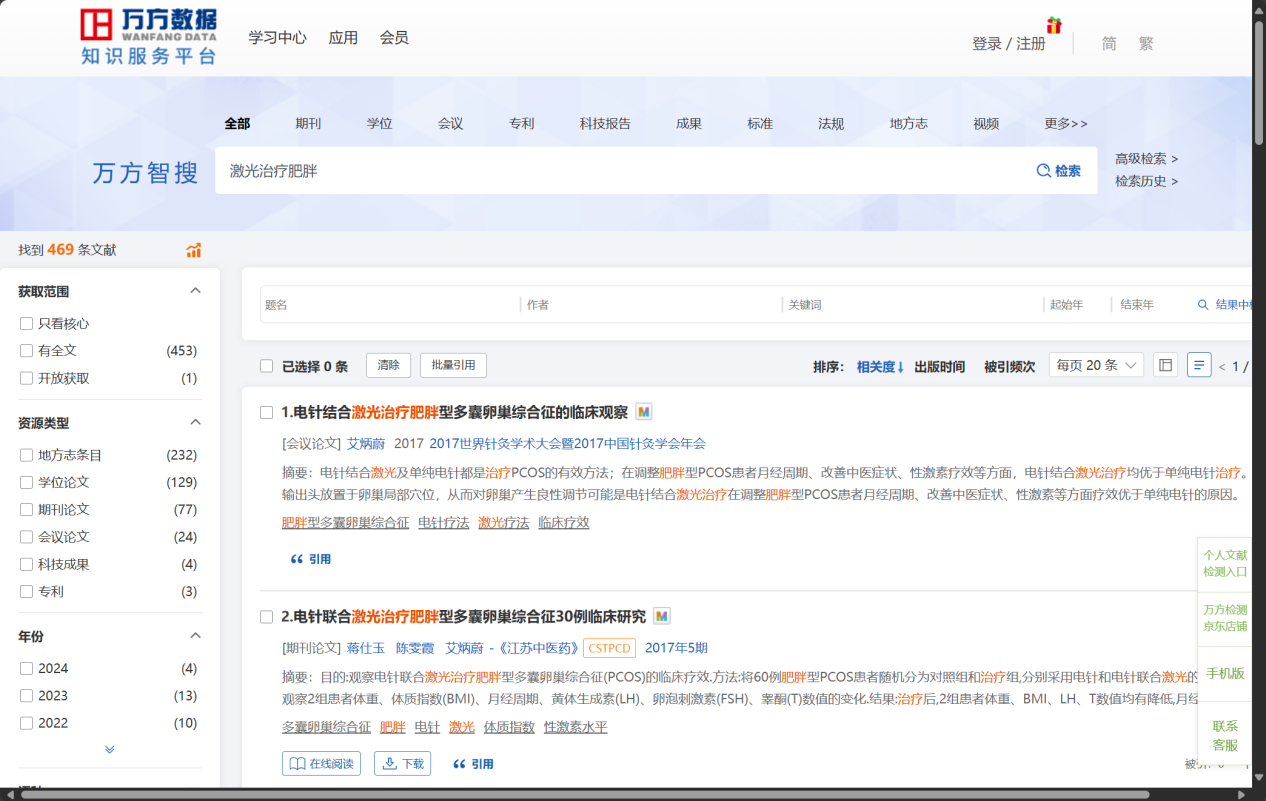

Supplement: Supplementary file 3 — Supplementary Material 3. S3. Search strategy and specific journals list. [file 12906_2025_4874_MOESM3_ESM.doc]
