# Supplementary material for: Effectiveness of photobiomodulation therapy in improving health indicators in obese patients: a systematic review and meta-analysis of RCTs
Source: BMC Complement Med Ther. 2025 Apr 11;25:133. doi: 10.1186/s12906-025-04874-2 (PMC11992763; doi:10.1186/s12906-025-04874-2)
Supplement: Supplementary file 4 — Supplementary Material 4. S4. BMI subgroup and sensitivity analysis. [file 12906_2025_4874_MOESM4_ESM.doc]

**Supplementary Material S4 BMI subgroup analysis and sensitivity analysis**


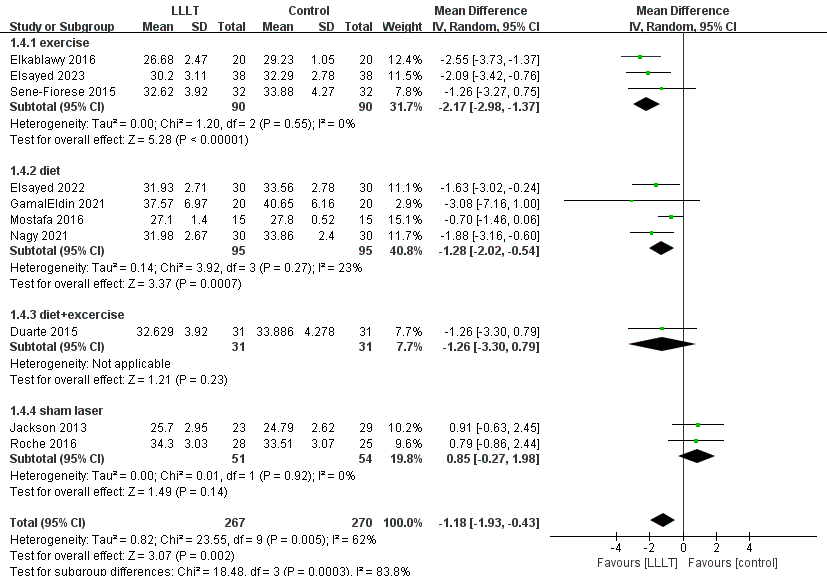


1. **BMI subgroup analysis based on different control group interventions**


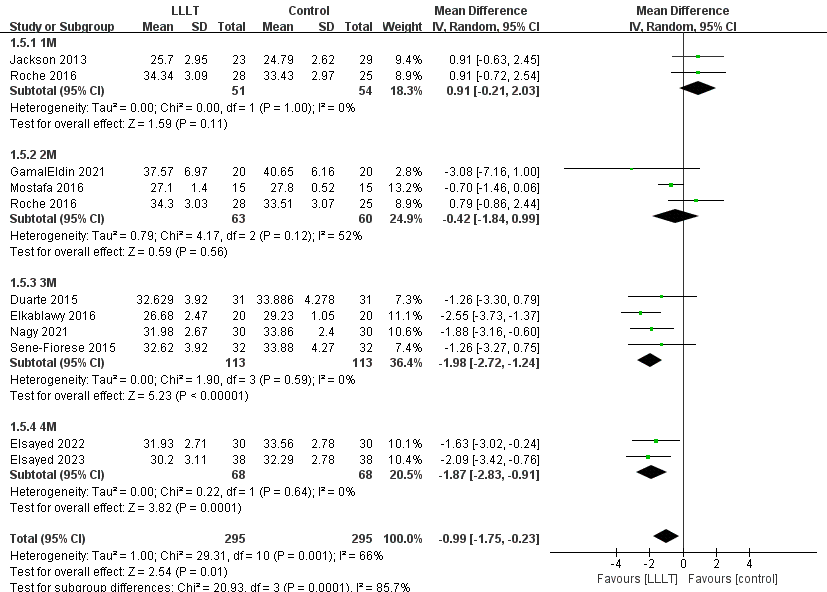


1. **BMI subgroup analysis based on different follow up time**


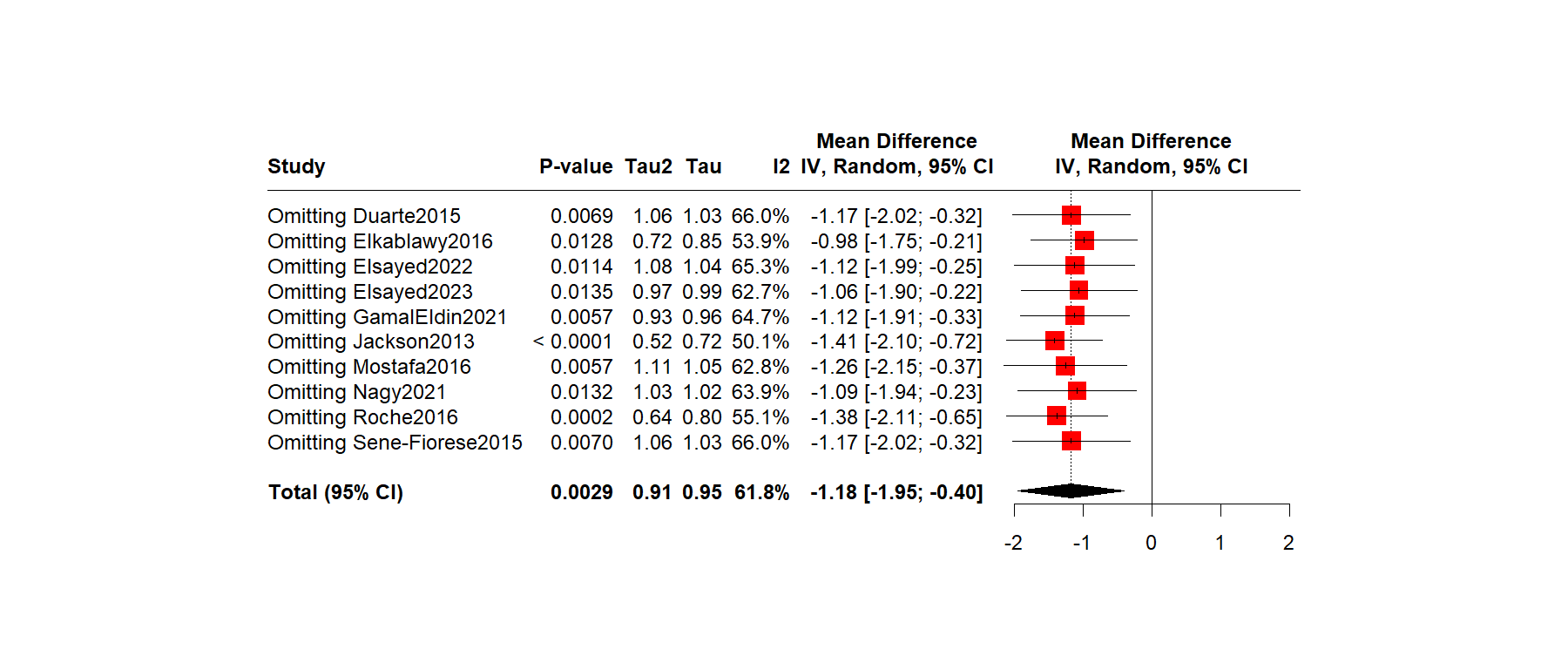
**3. BMI sensitivity analysis**
