# Supplementary material for: Effectiveness of photobiomodulation therapy in improving health indicators in obese patients: a systematic review and meta-analysis of RCTs
Source: BMC Complement Med Ther. 2025 Apr 11;25:133. doi: 10.1186/s12906-025-04874-2 (PMC11992763; doi:10.1186/s12906-025-04874-2)
Supplement: Supplementary file 5 — Supplementary Material 5. S5. Weight subgroup and sensitivity analysis. [file 12906_2025_4874_MOESM5_ESM.doc]

**Supplementary Material S5 Weight subgroup and sensitivity analysis**


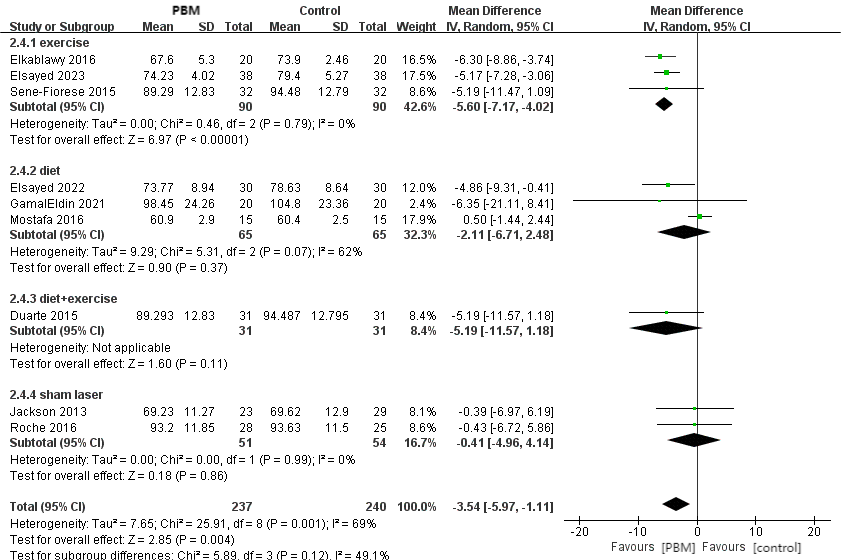


**
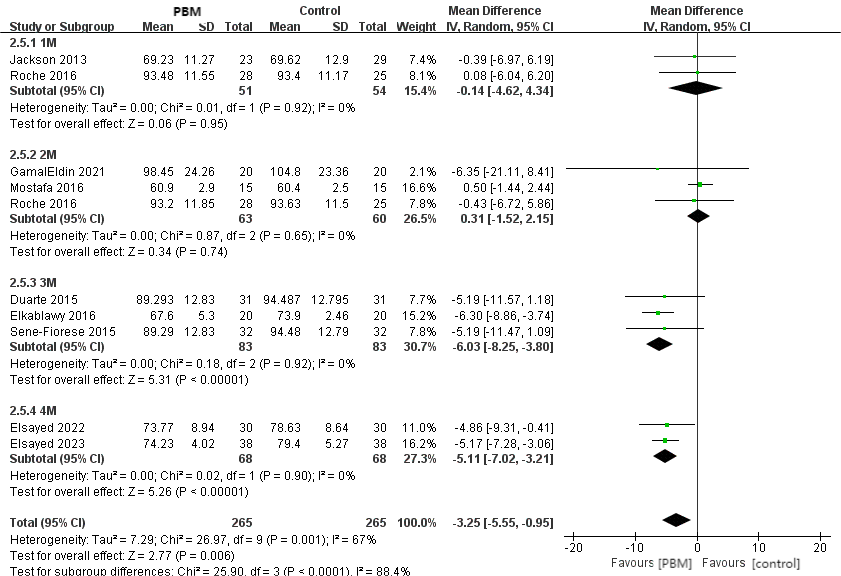
**

1. **Weight subgroup analysis based on different control group interventions**

**Weight subgroup analysis based on different follow up time**
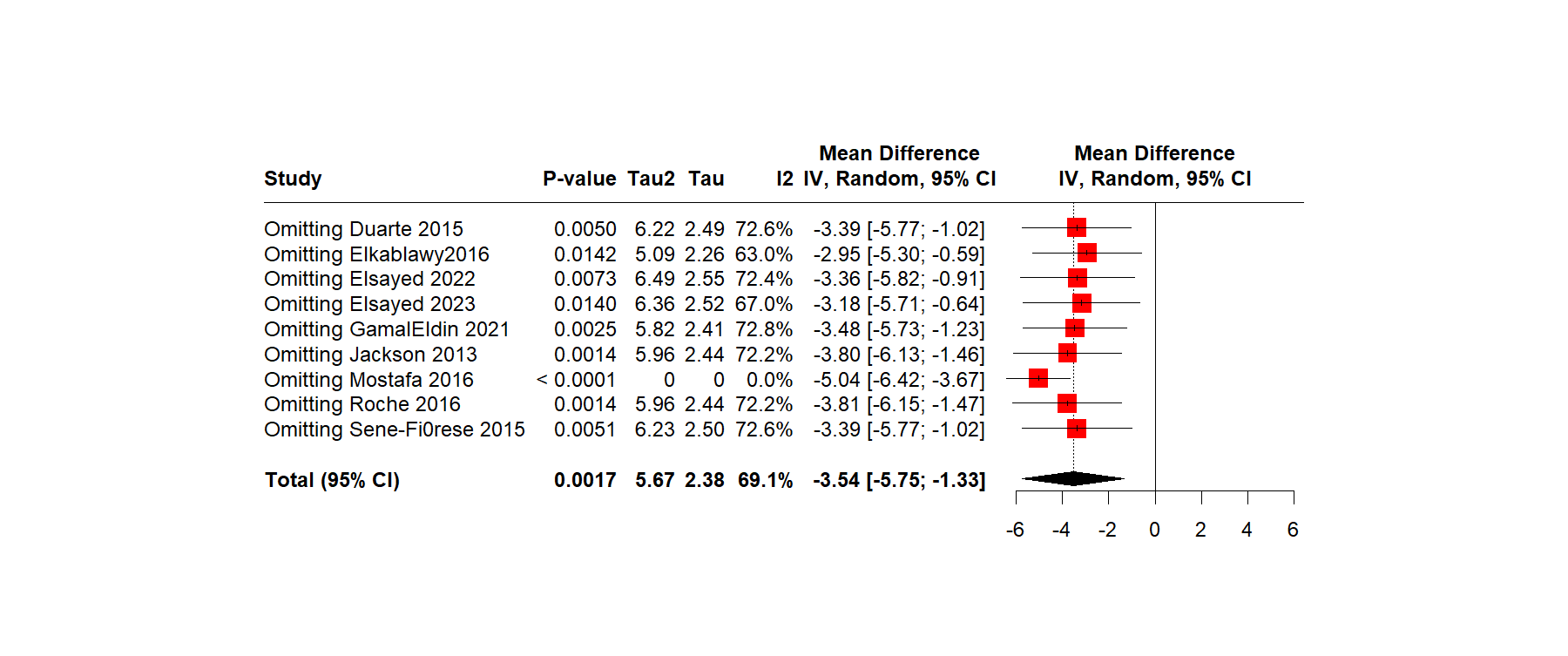


**3.Weight sensitiity analysis**


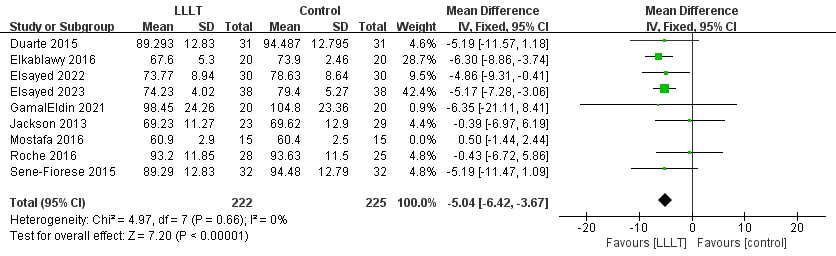


1. **Analysis after excluded Mostafa**
