# Supplementary material for: Effectiveness of photobiomodulation therapy in improving health indicators in obese patients: a systematic review and meta-analysis of RCTs
Source: BMC Complement Med Ther. 2025 Apr 11;25:133. doi: 10.1186/s12906-025-04874-2 (PMC11992763; doi:10.1186/s12906-025-04874-2)
Supplement: Supplementary file 6 — Supplementary Material 6. S6. Waistline subgroup and sensitivity analysis. [file 12906_2025_4874_MOESM6_ESM.doc]

**Supplementary Material S6 waistline subgroup and sensitivity analysis**

**
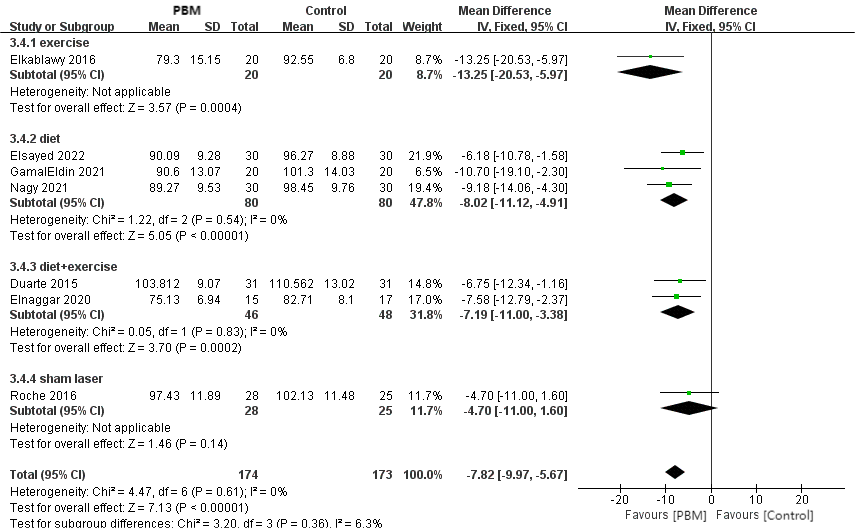
**

1. **Waistline subgroup analysis based on different control group interventions**
2.
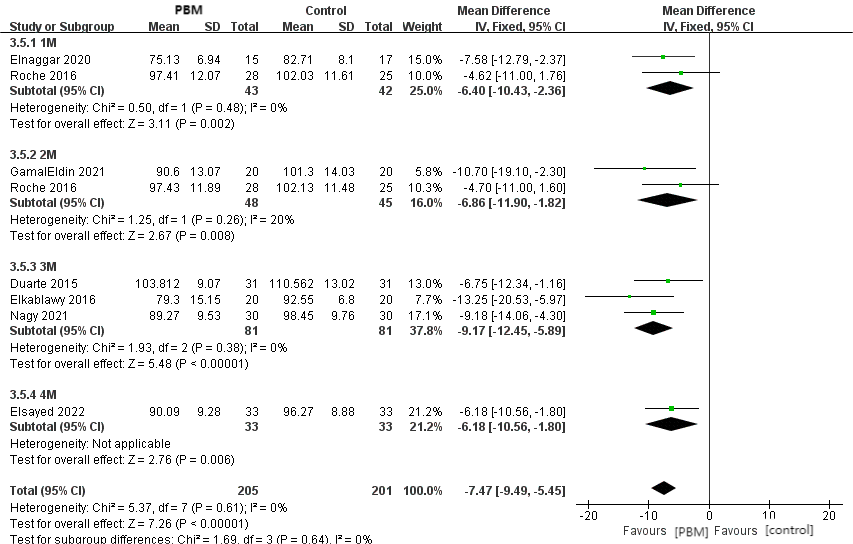
**Waistline subgroup analysis based on different follow up time**

**
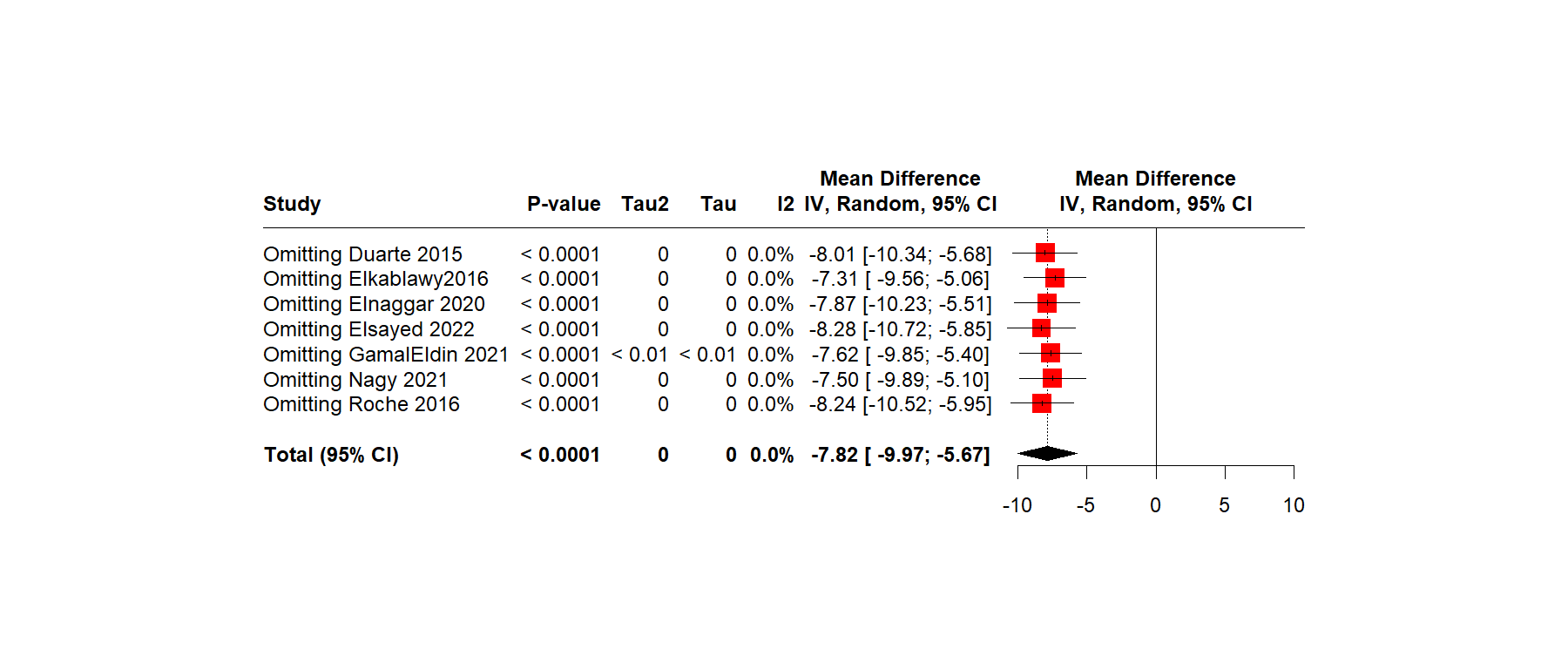
**

1. **Waistline sensitivity analysis**
