# Supplementary material for: Effectiveness of photobiomodulation therapy in improving health indicators in obese patients: a systematic review and meta-analysis of RCTs
Source: BMC Complement Med Ther. 2025 Apr 11;25:133. doi: 10.1186/s12906-025-04874-2 (PMC11992763; doi:10.1186/s12906-025-04874-2)

**Supplementary Material S7 Other outcomes**

1. ***WHR***

***
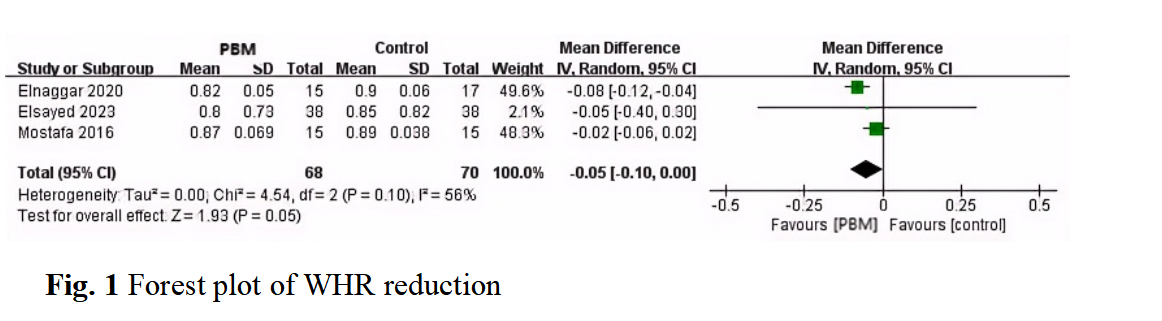
***

1. ***Fat mass percentage***


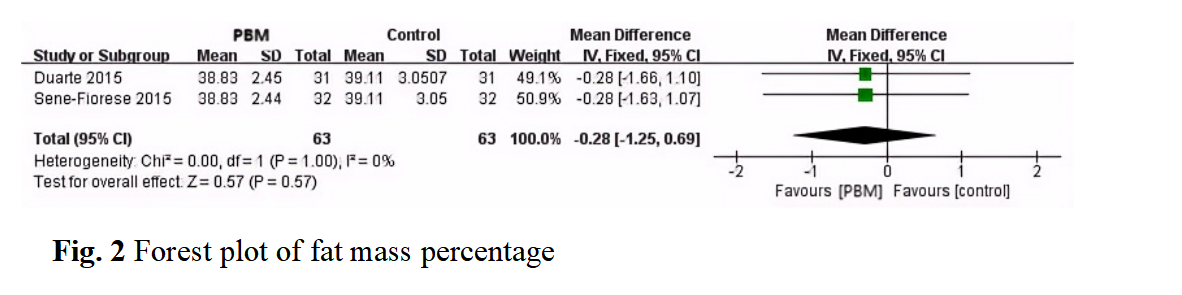


1. ***C-reactive protein***


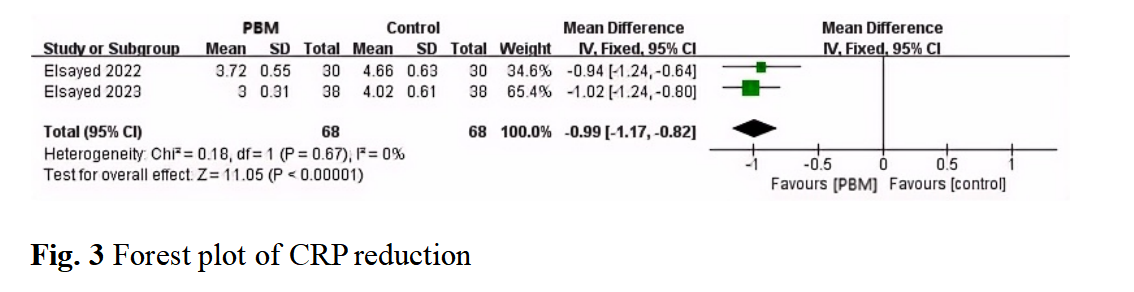


1. ***Total cholesterol***


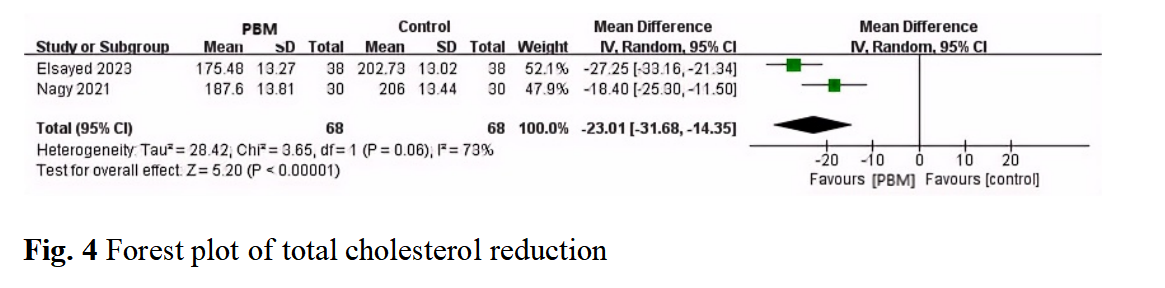


1. ***Insulin***


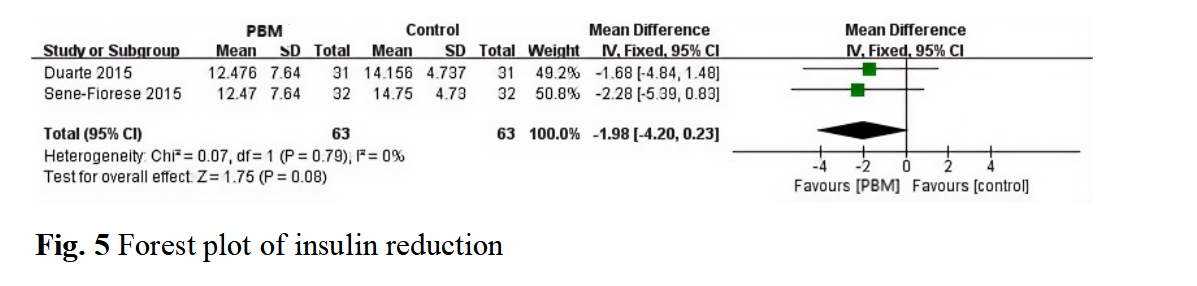


1. ***HOMA-IR***


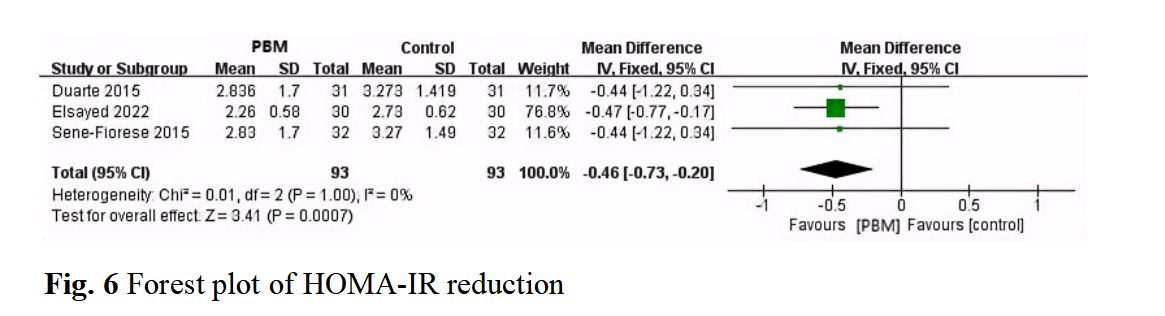

Supplement: Supplementary file 7 — Supplementary Material 7. S7. Other outcomes analysis. [file 12906_2025_4874_MOESM7_ESM.doc]
