# Supplementary material for: Effectiveness of photobiomodulation therapy in improving health indicators in obese patients: a systematic review and meta-analysis of RCTs
Source: BMC Complement Med Ther. 2025 Apr 11;25:133. doi: 10.1186/s12906-025-04874-2 (PMC11992763; doi:10.1186/s12906-025-04874-2)

**Supplementary Material S8 publication bias**

**Egger's Test Results for BMI:**

Linear regression test of funnel plot asymmetry

Test result: t = -0.24, df = 8, p-value = 0.8156

Bias estimate: -0.3598 (SE = 1.4926)


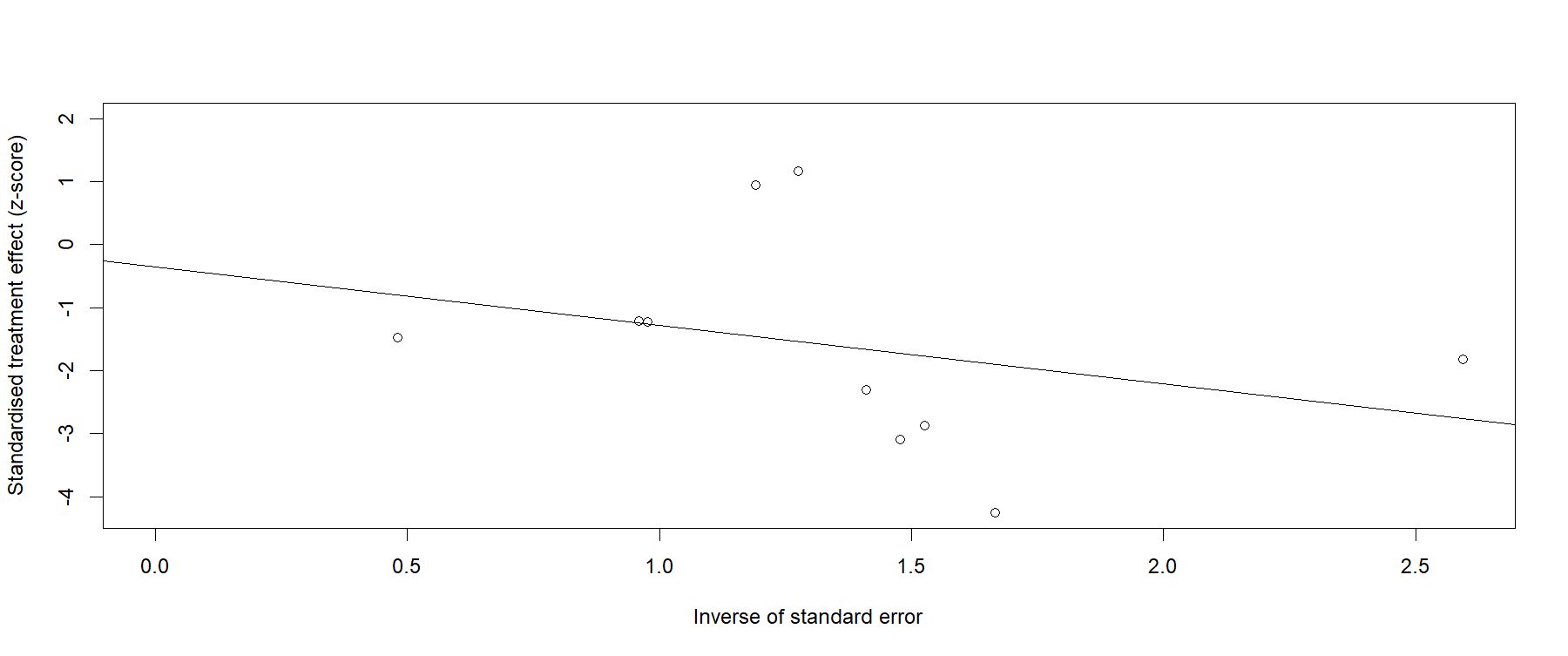


**Egger's Test Results for Weight:**

Linear regression test of funnel plot asymmetry

Test result: t = -0.40, df = 7, p-value = 0.6982

Bias estimate: -0.5000 (SE = 1.2374)


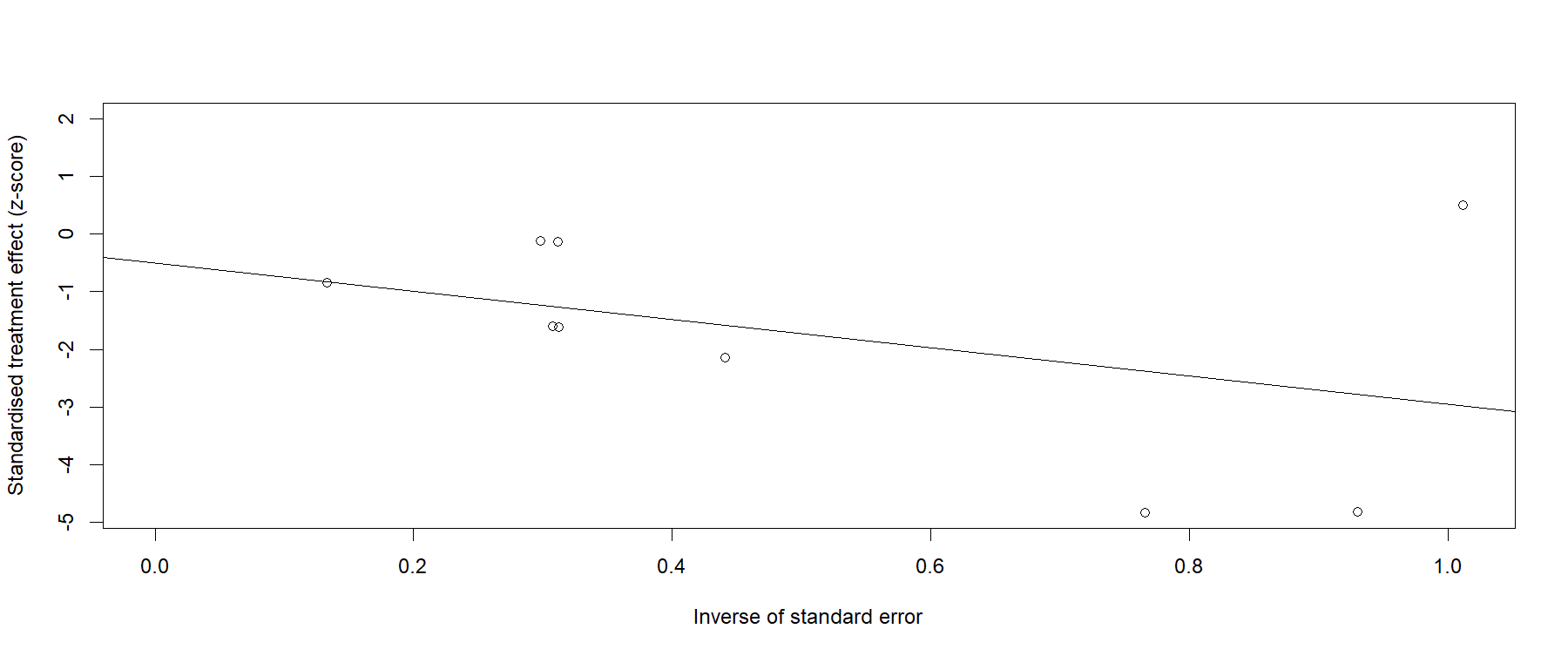


**Egger's Test Results for** **Waistline :**

Linear regression test of funnel plot asymmetry

Test result: t = -1.30, df = 5, p-value = 0.2494

Bias estimate: -2.1220 (SE = 1.6286)


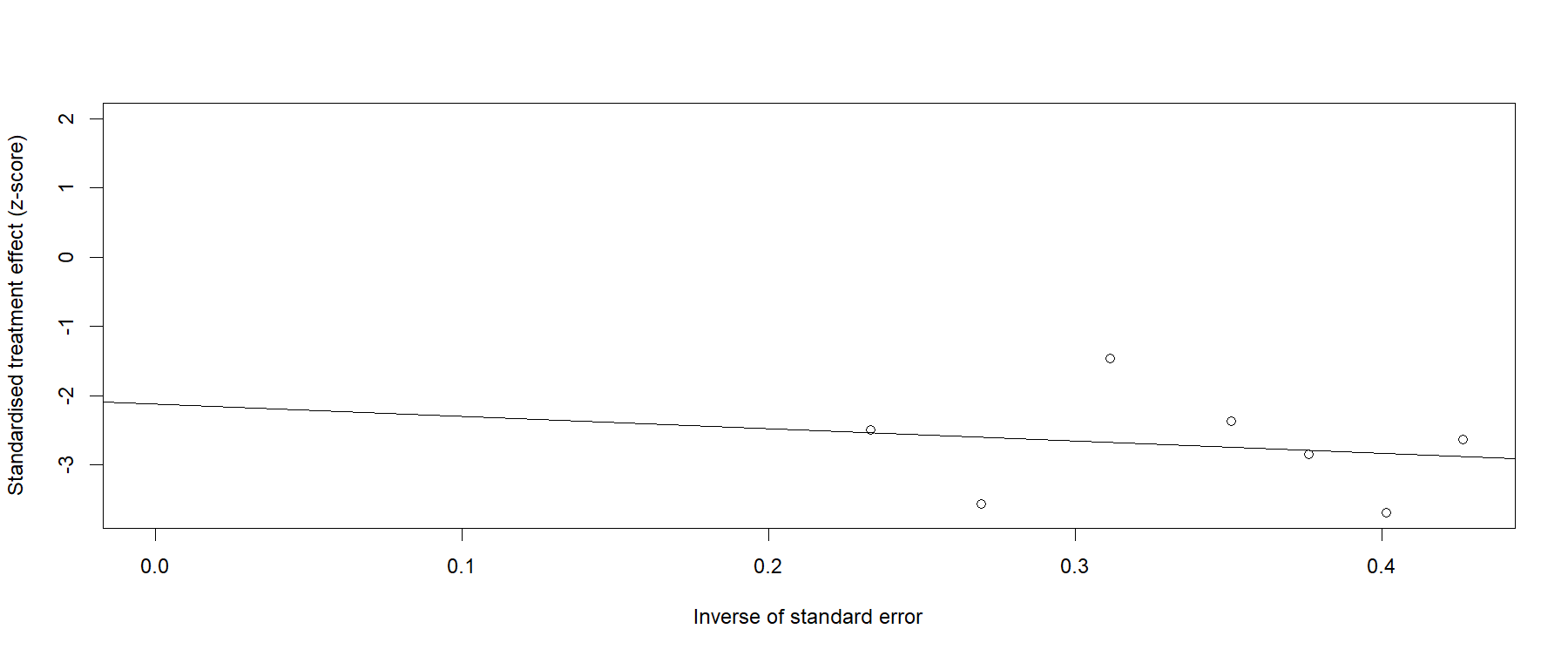

Supplement: Supplementary file 8 — Supplementary Material 8. S8. Publication bias. [file 12906_2025_4874_MOESM8_ESM.doc]
